# Mathematical and Computational Models of Biochemical Reactions and Cell Signaling—From Ordinary Differential Equations to Machine Learning

**DOI:** 10.3390/ijms27156839

**Published:** 2026-07-30

**Authors:** Grzegorz Matyszczak

**Affiliations:** Faculty of Chemistry, Warsaw University of Technology, Noakowski Street 3, 00-664 Warsaw, Poland; grzegorz.matyszczak@pw.edu.pl

**Keywords:** ordinary differential equations, machine learning, biochemical reactions, enzyme kinetics, cell signaling, systems biology, mathematical modeling

## Abstract

Cell signaling, and the biochemical reactions underlying it, are complex phenomena fundamental for control of cellular behavior in response to the incentives present in the cell’s direct environment. It is crucial for coordination of cell activities such as growth, differentiation, metabolism, and death. The aim of this review is to present mathematical and computational models of biochemical reactions and cell signaling pathways in an educational and comprehensive way, outlining broad aspects of modeling such as differential equations, and artificial intelligence and machine learning approaches. This review also discusses potential applications of mathematical and computational models of cell signaling and biochemical reactions in fields such as systems biology, personalized medicine (i.e., cancer treatment, neurodegenerative disease treatment), and identification of drug targets.

## 1. Introduction

**Figure 1:**
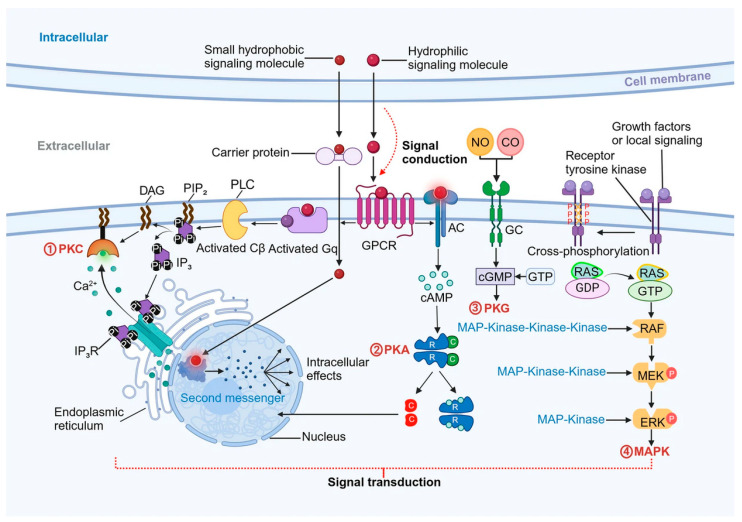
Schematic representation of cell signaling pathways. Signal is constructed through interaction of ligands with receptors, which converts ligands into intracellular signals. Transduction of these signals guarantee successful performance of various physiological functions affecting cell metabolism that contributes to the maintenance of organism’s homeostasis. Abbreviations: AC—adenylate cyclase, cAMP—cyclic adenosine monophosphate, cGMP—cyclic guanosine monophosphate, CO—carbon monoxide, DAG—diacylglycerol, ERK—extracellular-regulated protein kinases, GC—guanylate cyclase, GDP—guanosine-5′-diphosphate, GPCR—G-protein-coupled receptor, GTP—guanosine triphosphate, IP3—inositol trisphosphate, IP3R—inositol trisphosphate receptor, MAPK—mitogen-activated protein kinase, MEK—mitogen-activated extracellular signal-regulated kinase, NO—nitric oxide, Pi PIP2—phosphatidylinositol-4,5-bisphosphate, PKA—protein kinase A, PKC—protein kinase C, PKG—protein kinase G, PLC—phospholipase C. Reproduced under Creative Commons license from ref. [3] with no changes.

Cell signaling and biochemical reactions are crucial for regulating functions of cells in multicellular organisms [1,2,3]. Cell signaling depends on molecular signals which are transmitted via biochemical reactions. The latter are started by binding the extracellular ligand (e.g., hormone or growth factor) to specific receptors present on the surface of the cell (e.g., receptor tyrosine kinases (RTKs) or G-protein-coupled receptors (GPCRs)) (see Figure 1) [4,5,6,7,8,9]. Such binding results in changes in the structure of the receptor causing a cascade of intracellular incidents—a signal is transmitted into the targeted cell. Various intracellular messengers (e.g., phosphoinositides, Ca^2+^ ions or cAMP) are involved in the amplification and transmission of the signal within the cell [10,11,12,13,14]. They modulate the activities of enzymes that result in, for example, certain cellular responses (e.g., proliferation or death) or influenced expression of genes.

**Figure 2:**
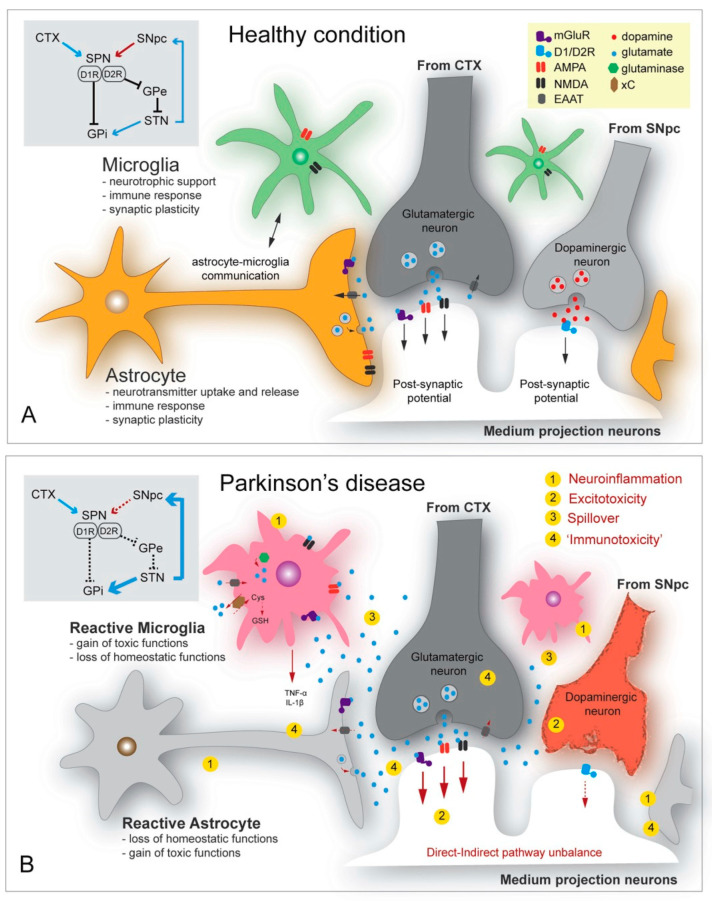
Cell signaling in healthy (**A**) and Parkinson’s disease (**B**) conditions. Abbreviations: AMPA—alpha-amino-3-hydroxy-5-methyl-4-isoxazolepropionic acid; CTX—cerebral cortex; Cys—cysteine; EEAT—excitatory amino acid transporter; Gpe—external segment of the globus pallidus; Gpi—internal segment of the globus pallidus; GSH—glutathione; mGluR—metabotropic glutamate receptor; NMDA—N-methyl-D-aspartate; Snpc—substantia nigra pars compacta; SPN—spiny projection neuron; STN—subthalamic nucleus; xC—cysteine–glutamate exchange system. Reproduced under Creative Commons license from ref. [23] with no changes.

**Figure 3:**
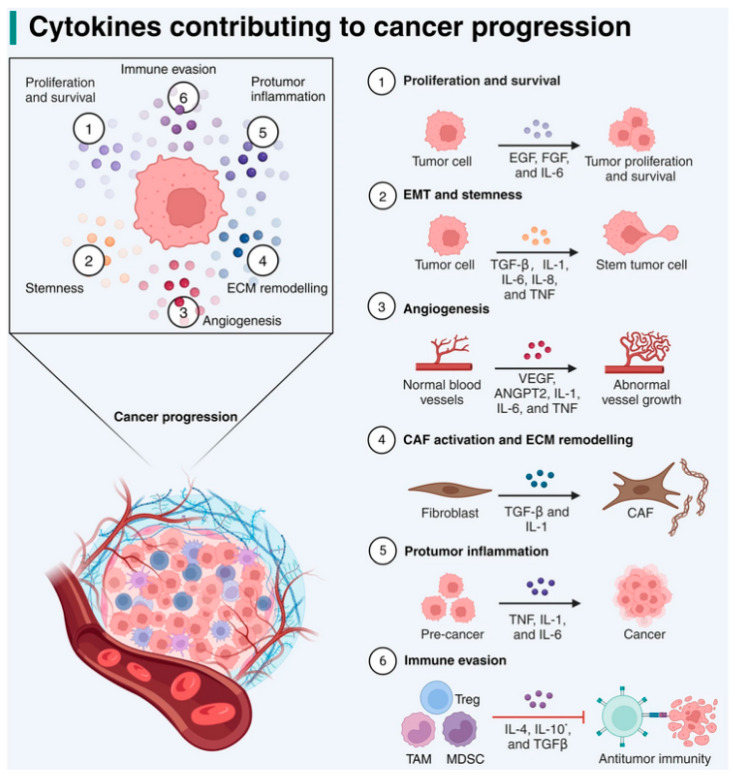
Schematic representation of multifaceted actions (proliferation, epithelial–mesenchymal transition (EMT), angiogenesis, fibroblast activation, protumor inflammation, and immune suppression) of cytokines in cancer disease. Reproduced under Creative Commons license from ref. [27] with no changes.

Changes in the environment of individual cells may include the appearance of pathogens, hormones, and/or growth factors. The complex network of signaling pathways ensures the adequate reaction of cell to the stimuli in its surroundings [15,16,17,18,19]. Disrupted cell signaling is considered to be a reason underlying many diseases like cancer, autoimmune disorders, and neurodegenerative conditions (see Figure 2 and Figure 3) [20,21,22,23,24,25,26]. The development of novel therapeutic strategies for such diseases requires better understanding of cell signaling mechanisms [27,28]. Insights into cell signaling mechanisms are possible thanks to the biochemical methods; however, computational methods are also very useful due to the complex nature of the phenomena [29,30,31]. The application of computation in biology significantly improved our understanding of cell signaling. For example, computational models may be used to identify drug targets within the cell signaling pathways as in the case of development of new inhibitors of abnormal signaling in cancer cells [32,33]. Scientists used various mathematical models including, but not limited to, deterministic models such as ordinary differential equations, stochastic models such as stochastic differential equations, and machine learning algorithms [34,35,36,37]. Computational models are possible for predicting cell signaling pathway behavior under novel circumstances, such as pharmacological or genetic interventions [38,39,40,41,42]. Cell signaling networks are complex systems and computer models allow for the identification of critical nodes within them [43,44,45,46]. Other complex phenomena within signaling networks, that are being modeled using bifurcation analysis, include periodic and bistable behaviors which are crucial for regulation of the cell cycle (oscillations) and for cell death (bistability) [47,48,49,50].

Synthetic biology is a discipline that especially profits from the development of computational modeling which simplifies the construction of artificial signaling networks for applications in, among others, gene therapies, programming of cell behavior, and biosensors [51,52,53,54]. Thanks to that it is possible to create specially engineered cells with the aim to modulate disease pathways directly within the patient’s organism [55]. Computational methods, merged with sufficient experimental data, may be used to predict the action of engineered cells in a living organism under various pathological and physiological conditions, allowing for the minimization of side effects and maximization of therapy efficiency [56,57]. 

**Figure 4:**
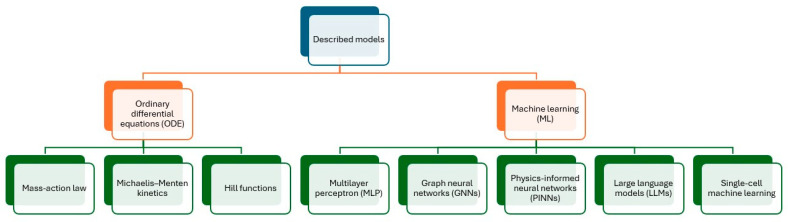
Outline of topics covered in the review.

The aim of this review is to provide a comprehensive and educative framework of computational and mathematical models, based on ordinary differential equations and machine learning (see Figure 4), used in modeling of the cell signaling networks and biochemical reactions. The main goals are divided into three categories:

- to present the most important models of cell signaling and biochemical reactions;
- to present methods of analysis of network topology and complex behavior of models, including bistability and oscillations;
- to present practical and real-life applications of mathematical and computational models of cell signaling and biochemical reactions.

## 2. Differential Equations

The oldest and most known approach in mathematical modeling of biological phenomena, including biochemical reactions and cell signaling, is based on differential equations which allow for simulation of changes in continuous variables (most often concentrations) over time [58,59,60,61].

### 2.1. Ordinary Differential Equations

Models based on ordinary differential equations (ODEs) are used to describe continuous changes in concentrations of chemical individuals (in case of biochemical reactions) or signaling molecule (in case of cell signaling). ODE-based models are deterministic in nature which means that the exact values of dependent variables (mostly concentrations, sometimes temperature and other quantities) may be predicted knowing initial conditions with sufficient accuracy [60,62]. This type of model is especially useful for systems with a large number of molecules and negligible randomness [60,61]. Additionally, complex reaction networks may be easily transformed into ODE-based models using simple mass-action law [63,64]. ODE-based models have been, for example, successfully utilized for the purpose of modeling of cell cycle kinetics [65,66]. Having once fitted an ODE model, one may use it to predict the behavior of an investigated system in novel situations, such as a changed cellular environment or mutations [67,68]. The predictions of the model may be used for its improvement—after comparing with experimental data—or to propose new hypotheses. Ordinary differential equations may be generally easily solved using analytical techniques (but not always) and numerical methods (such as the Runge–Kutta algorithm) [69,70,71]. Nowadays, ODE-based models are viewed as fundamental tools in mathematical and computer biology due to the balance between their relatively low computational cost and relatively high complexity of modeled phenomena.

#### 2.1.1. The Mass Action Law

The law of mass action is the most basic approach for obtaining kinetic characterization of a system of (bio)chemical reactions, which may also be extended to simulations of population dynamics or the spread of various diseases [72,73,74]. This law states that for a spatially homogeneous system the rates of involved elementary chemical reactions are proportional to the products of concentrations of reacting species [72]. The proportionality constant is known as the rate constant. For a (bio)chemical reaction, it is dependent on quantities such as temperature (through the Arrhenius equation), solvent constituent matrix in the system, and pH of the system [75,76,77]. Due to this, in biochemical experiments, typically reaction conditions are kept constant to avoid unnecessary complication caused by this dependence. 

To see the mass action law working, let us consider a system of m independent elementary (bio)chemical reactions involving n distinct reactants. Such a system may be written as the following equation:

(1) $$\sum_{i=1}^{n}s_{i,j}X_{i}\to \sum_{i=1}^{n}p_{i,j}X_{i}\;j=1,\ldots ,m$$

where X_i_ denotes *i*-th reactant, s_i,j_ and p_i,j_ denote stoichiometric coefficients when X_i_ appears as substrate and product, respectively.

Now, assuming that the system of interest is closed and homogeneous, one may write down ordinary differential equations describing the change in concentration (C_i_) over time of each reactant:

(2) $$\frac{{dC}_{i}}{dt}=\sum_{j=1}^{m}r_{i,j}=r_{i}i=1,\ldots ,n$$

where r_i,j_ denotes the rate of reaction of *i*th reagent in *j*th reaction. These rates may be expressed using the law of mass action as a product of concentrations of reagents and kinetic parameters k_j_:

(3) $$r_{i,j}=\left(p_{i,j}-s_{i,j}\right)k_{j}\prod_{i=1}^{n}C_{i}^{s_{i,j}}\;i=1,\ldots ,nj=1,\ldots ,m$$

Although some chemical reactions may be considered reversible and apparently unsuitable for the scheme described above, they may be divided into two independent irreversible steps which may be included in Equation (1).

#### 2.1.2. Michaelis–Menten Kinetics

As an example of the application of the mass action law, one of the most simple enzymatic reactions will be considered. Its general scheme is presented below:

(4) $$S+E\begin{array}{c} \overset{k_{1}}{\to }\\ \underset{k_{-1}}{\leftarrow } \end{array}C\overset{k_{2}}{\to }E+P$$

This scheme involves a reversible reaction of a substrate (S) with an enzyme (E) giving an intermediate substrate–enzyme complex (C) which transforms to product (P) restoring the enzyme at the same time in an irreversible reaction. Now, using the law of mass action, it will be shown how Michaelis–Menten kinetics, used commonly in enzymology, results from this relatively simple equation [78,79,80]. Using the law one may write a system of ordinary differential equations describing the changes in concentrations of reagents over time, which after some simple transformations may be written as:

(5) $$\frac{d\left[S\right]}{dt}=k_{1}\left(-\left({\left[E\right]}_{0}-\left[C\right]\right)\left[S\right]+K_{S}\left[C\right]\right)$$

(6) $$\frac{d\left[C\right]}{dt}=k_{1}\left(\left({\left[E\right]}_{0}-\left[C\right]\right)\left[S\right]-K_{M}\left[C\right]\right)$$

(7) $$\frac{d\left[P\right]}{dt}=k_{2}\left[C\right]$$

This form is obtained using the following conservation law for the enzyme:

(8) $$\left[E\right]\left(t\right)={\left[E\right]}_{0}-\left[C\right]\left(t\right)$$

and incorporating two novel constants: K_S_—the equilibrium constant of the formation-dissociation of substrate–enzyme complex, and K_M_—the so-called Michaelis–Menten constant:

(9) $$K_{S}=k_{-1}k_{1}$$

(10) $$K_{M}=\left(k_{-1}+k_{2}\right)k_{1}$$

Such a system (Equations (5)–(7)) may be solved numerically using certain initial conditions (so-called initial value problem), for example:

(11) $$\left[S\right]\left(0\right)={\left[S\right]}_{0}$$

(12) $$\left[C\right]\left(0\right)=0$$

(13) $$\left[P\right]\left(0\right)=0$$

However, the exact solution to the system may be obtained after adopting the quasi-steady state approximation regarding the concentration of the substrate–enzyme complex [81,82,83]:

(14) $$\left[C\right]\left(t\right)=\frac{{\left[E\right]}_{0}\left[S\right]\left(t\right)}{K_{M}+\left[S\right]\left(t\right)}$$

Thanks to that the systems of ODEs, Equations (5)–(7) may be rewritten in a form which may be solved analytically:

(15) $$\frac{d\left[P\right]}{dt}=\frac{-d\left[S\right]}{dt}=\frac{v_{max}\left[S\right]}{K_{M}+\left[S\right]}$$

where v_max_ is the maximal reaction rate:

(16) $$v_{max}=k_{2}{\left[E\right]}_{0}$$

#### 2.1.3. Complex Enzyme Kinetics and Hill Functions

The described above enzymatic reaction, resulting in Michaelis–Menten kinetics, has the most simple mechanism. Many enzymatic reactions with far more complicated mechanisms are present in enzymologic studies. The following reaction scheme represents a general inhibition mechanism [84,85]:

(17) $$S+E+I\begin{array}{c} \overset{k_{1}}{\to }\\ \underset{k_{-1}}{\leftarrow } \end{array}ES+I\overset{k_{2}}{\to }E+I+P$$

(18) $$S+E+I\begin{array}{c} \overset{k_{3}}{\to }\\ \underset{k_{-3}}{\leftarrow } \end{array}EI+S\begin{array}{c} \overset{k_{5}}{\to }\\ \underset{k_{-5}}{\leftarrow } \end{array}ESI\begin{array}{c} \overset{k_{-4}}{\to }\\ \underset{k_{4}}{\leftarrow } \end{array}ES+I$$

where E, S and P symbols have the same meaning as above, and I is the inhibitor. ES and EI are complexes of an enzyme with substrate or inhibitor, respectively. ESI denotes the complex of enzyme, substrate, and inhibitor. The kinetics of this mechanism is described by large system of ODE and is much more computationally demanding than the simple Michaelis–Menten model. However, under quasi-steady-state approximation one may obtain a similar equation as above:

(19) $$\frac{d\left[P\right]}{dt}=\frac{-d\left[S\right]}{dt}=\frac{\tilde{v_{max}}\left[S\right]}{\tilde{K_{M}}+\left[S\right]}$$

In this equation v_max_ and K_M_ are replaced with their apparent versions expressed by following equations:

(20) $$\tilde{v_{max}}=\frac{v_{max}}{\left(1+\frac{\left[I\right]}{K_{SI}}\right)}$$

(21) $$\tilde{K_{M}}=K_{M}\frac{\left(1+\frac{\left[I\right]}{K_{I}}\right)}{\left(1+\frac{\left[I\right]}{K_{SI}}\right)}$$

A very similar equation, but derived experimentally, was proposed by Hill who observed a typical sigmoidal dependence of the rate of product formation with regard to the concentration of the substrate [86,87,88]:

(22) $$\frac{d\left[P\right]}{dt}=\frac{-d\left[S\right]}{dt}=\frac{v_{max}{\left[S\right]}^{n}}{K_{M}^{n}+{\left[S\right]}^{n}}$$

**Figure 5:**
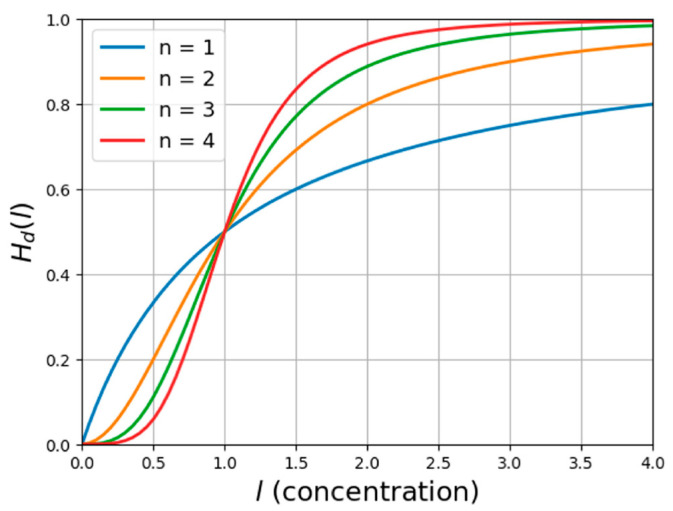
Sample plots of the Hill function taking v_max_ and K_M_ equal to 1 for distinct integer Hill coefficient values. Reproduced under Creative Commons license from ref. [90] with no changes.

In the above equation n denotes the so-called Hill coefficient. This parameter may adopt non-integer values in general [89,90,91]. However, an integer value of the Hill coefficient (see Figure 5) may have a mechanistic meaning as the number of ligand (L) molecules that binds with enzyme/receptor (R), according to the following reaction scheme [89,90,91]:

(23) $$R+nL\begin{array}{c} \overset{k_{1}}{\to }\\ \underset{k_{-1}}{\leftarrow } \end{array}{RL}_{n}$$

Catalytic reactions may be broken down into a set of elementary steps, including the formation and dissociation of an enzyme–substrate complex, formation of products, and potential isomerisation of various intermediates. Using the mass action law one may obtain a system of ordinary differential equations which may exhibit nonlinearity and multidimensionality [92,93,94]. Direct numerical solving of such systems may be computationally demanding; thus, methods for their simplification (e.g., the quasi-steady state approximation) and/or other techniques of simulation (e.g., machine learning) are necessary [92,93,94,95]. On the other hand, such models may be very useful for the scaling up of enzymatic reactions from a laboratory to industrial scale [96].

#### 2.1.4. Examples of Applications of ODE-Based Models for Modeling of Biochemical Reactions and Cell Signaling

##### **ODE-Based Modeling of AMPK and mTOR Crosstalk in Glutamatergic Synapse Calcium Signaling** 

**Figure 6:**
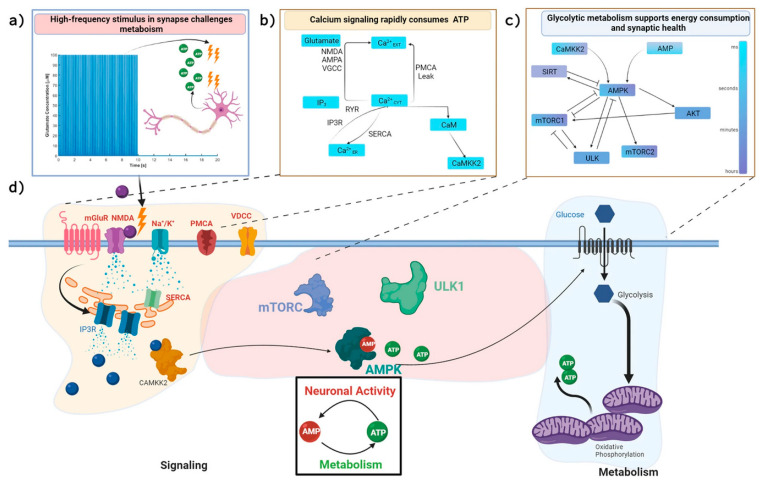
A schematic representation of synaptic signaling: (**a**) glutamate stimulus as high-frequency synaptic signaling favors allocation of larger ATP quantities for restoring resting potential of ions, (**b**) schematic representation of calcium signaling rapidly consuming ATP, (**c**) schematic representation of glycolytic metabolism, (**d**) summary of investigated pathways. Reproduced under Creative Commons license from ref. [97] with no changes.

AMP-activated protein kinase (AMPK) and mammalian target of rapamycin (mTOR) are two protein kinases that are important in metabolic regulation and cell fate which are activated during synaptic signaling (see Figure 6) [97]. Leung and Rangamani constructed a computational model of this signaling allowing for investigation of the influence of glutamate stimulus frequency on the dynamics of AMPK and mTOR [97]. The high-frequency oscillations of glutamate concentration stimulates neurons to allocate more ATP needed for the restoration of resting potentials of ions, like Ca^2+^ [97]. This may be challenging for the neurons capacity of energy production. The ATP is produced in dendritic mitochondria via glycolysis and oxidative phosphorylation [97]. The higher AMP to ATP ratio promotes phosphorylation of AMPK which subsequently promotes the production of ATP and, at the same time, influences mTORC1 and mTORC2 kinases in the insulin-signaling cascade [97]. This, on the other hand, affects protein translation and synaptic plasticity [97]. 

The model presented by Leung and Rangamani has three different modules: kinase activation, metabolism, and calcium signaling [97]. The consumption of ATP resulting in the activation of AMPK was a main component linking all three modules [97]. ATP consumption is treated using a simple model representing glycolysis, oxidative phosphorylation, and energy consumption. Authors adopted also a literature model of AMPK and mTOR activation in skeletal muscle downstream of activation of insulin receptor for modeling of the kinase activation [97]. Calcium dynamics is crucial for the induction of synaptic plasticity. Authors used well-established literature models of neuronal calcium [97]. An ODE-based approach required specification of all incomes and outcomes (i.e., fluxes) of Ca^2+^ ions. Authors presented following two ordinary differential equations for the overall calcium dynamics:

(24) $$\frac{d\left[{Ca}_{C}\right]}{dt}=J_{RYR}+J_{IP3}-J_{SERCA}+J_{PM}+J_{ER,Leak}-J_{Buff}$$

(25) $$\frac{d\left[{Ca}_{ER}\right]}{dt}=-J_{RYR}-J_{IP3}+J_{SERCA}-J_{ER,Leak}-J_{Buff}$$

in which Ca_C_ stays for the Ca^2+^ concentration in the cytosol, and Ca_ER_ stays for the Ca^2+^ concentration in the endoplasmic reticulum (ER). J denotes fluxes of Ca^2+^ cations: J_RYR_—from ryanodine receptors (RyR), J_IP3_—from IP3 receptor (IP3R), J_SERCA_—from SERCA pump, J_PM_—from plasma membrane calcium-aptase, J_ER,leak_—from leakage of Ca^2+^ in ER, and J_Buff_ and J_ER,Buff_—from buffering of the cytosol and ER, respectively [97]. 

A similar procedure was applied to the concentrations of AMP, ADP, and ATP molecules resulting in following ordinary differential equations:

(26) $$\frac{d\left[{ATP}_{C}\right]}{dt}=J_{Glyc}+J_{OP}-J_{AK}-J_{CK}-2J_{ATPCA}+J_{AMPK}$$

(27) $$\frac{d\left[{ADP}_{C}\right]}{dt}=-J_{Glyc}-J_{OP}+2J_{AK}+J_{CK}+2J_{ATPCA}$$

(28) $$\frac{d\left[{AMP}_{C}\right]}{dt}=-J_{AK}-J_{AMPK}$$

in which AMP_C_, ADP_C_, and ATP_C_ denote concentrations of nucleotides in cytosol. The following fluxes were incorporated: J_Glyc_—glycolysis, J_OP_—oxidative phosphorylation, J_AK_—adenylate kinase, J_CK_—creatine kinase, J_ATPCA_—flux representing ATP consumption by SERCA and PMCA pumps, and J_AMPK_—flux originating from AMPK activation [97]. 

**Figure 7:**
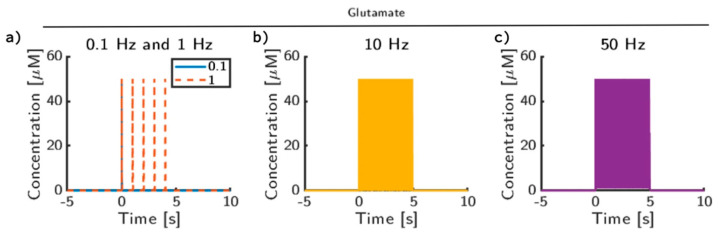
Plots of glutamate signals of various frequency: (**a**) 0.1 Hz, (**b**) 1 Hz, (**c**) 50 Hz. Reproduced under Creative Commons license from ref. [97] with no changes.

Authors used a Hill-type function for the dependence of oxidative phosphorylation (J_OP_) on Ca^2+^ concentration in cytosol and assumed a reliability of the mass action law for the production and consumption of ATP using kinetic parameters taken from literature [97,98]. The release of glutamate from the presynaptic cell into the synapse was modeled as a series of step functions, presented in Figure 7 [97]. The kinases AMPK and mTOR were coupled within an intricate feedback loop which involved many other protein kinases [97]. The authors adopted the signaling pathway for mTOR and AMPKs from other studies and integrated it with the metabolic activation of AMPK kinase through the ratio of AMP to ATP [97,99].

**Figure 8:**
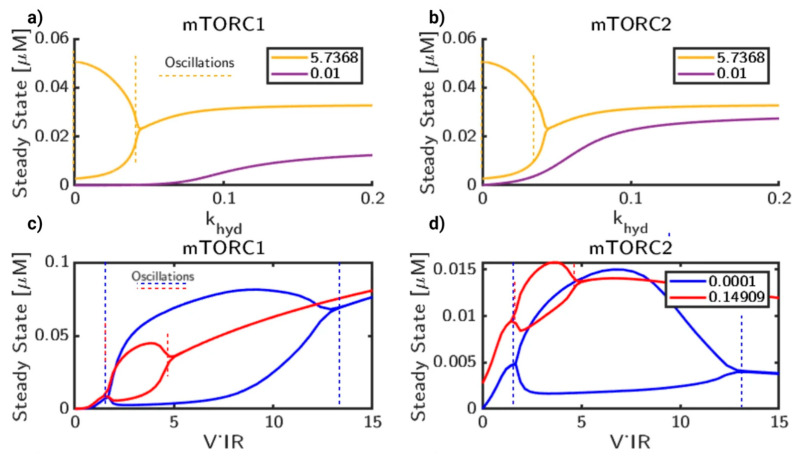
Plots representing the dependence of steady states of mTORC1 and mTORC2 depending on: (**a**,**b**) k_hyd_ parameter keeping V_IR_ parameter constant (5.7368 mM/s for yellow color, 0.01 mM/s for purple color); (**c**,**d**) V_IR_ parameter keeping k_hyd_ parameter constant (0.0001 mM/s for blue color, 0.149 mM/s for red color). Reproduced under Creative Commons license from ref. [97] with no changes.

The authors ended up with a model incorporating 60 species, 163 parameters, and 55 ordinary differential equations. A MATLAB ode15s built-in solver was used to solve the system of ordinary differential equations [97]. Due to the relatively large size and complexity of the model, potentially exhibiting nonlinear behavior, changes in a small number of parameters may lead to big changes in kinase concentrations. For that reason authors applied global sensitivity analysis using Latin Hypercube Sampling method [97]. For each set of parameter values authors simulated the steady state concentrations of AMPK, AKT, mTORC1, and mTORC2, and compared them with the default values. Then they performed a partial correlation analysis to determine the linear relationship between the parameters and output quantities [97]. Thanks to the applied ODE-based modeling, it was found that the increasing of the glutamate stimulus frequencies over 10 Hz perturb mTOR and AMPK oscillations at higher magnitudes by up to 36% [97]. Authors were also able to study the effect of crosstalk between calcium signaling and insulin receptor on mTOR and AMPK activation predicting multistability in a signaling network (see Figure 8) [97].

##### **Computational Model for Cell Energy Balance and Metabolism Based on Interactions Among mTORC, AMPK, and SIRT** 

**Figure 9:**
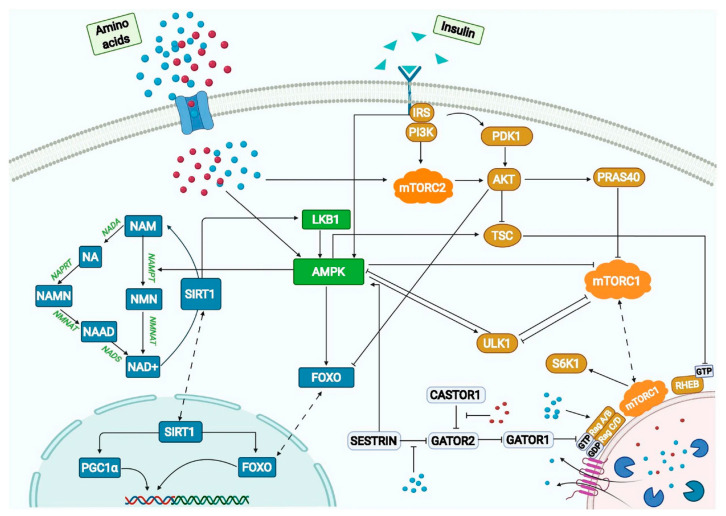
Representations of main metabolic pathways included in Sadria and Leyton model. Brown color indicates the insulin/IGF-1/mTOR signaling pathway, blue color indicates the Preiss–Handler and salvage pathways, green color indicates AMPK coupling in two major pathways. Blue circles indicate leucine, red circles indicate arginine, and green triangles indicate insulin. White rounded rectangles indicate amino acid sensors. Biochemical reactions are represented by solid arrows while protein translocation is represented by dashed arrows. Reproduced under Creative Commons license from ref. [100] with no changes.

The ability of cell metabolism adaptation to signals from their surroundings is crucial for their survival [100]. A deficit of such mechanisms in tissues is linked with diseases (e.g., cancer, neurological conditions) and aging [100]. mTOR and AMPK are particular examples of proteins playing an important role in cell adaptation and energy balance management in mammals. Sadria and Layton developed a ODE-based model of metabolic signaling pathways to investigate the interactions of mTORC and AMPK signaling networks and environmental stimuli with special focus on implications on lifespan and health-span [100]. Their model included the insuling/IGH-1 pathway (coupling abundance of energy and nutrient to the performance of cell division and growth), mTORC1 and the amino acid sensors, the Preiss–Handler and salvage pathways (regulating NAD+ metabolism), AMPK as an energy sensor, and FOXO and PGC-1α as transcription factors [100]. Figure 9 presents a pathway representation of a metabolism model [100]. 

The model used by Sadria and Leyton was mainly based on the mouse C2C12 myoblast cell line [100]. The authors developed a model consisting of a large system of ordinary differential equations and algebraic equations with 175 parameters. Values of these parameters were taken from the published literature or obtained by fitting to experimental data. The main novelty of this study was the explicit representation of the influence of sestrin2 and leucine on the regulation of mTORC1 through the following equation [100]:

(29) $$\begin{array}{c} \frac{d}{dt}\left[mTORC1pS2448\right]=-k_{TSC}\left[mTORC1pS2448\right]\left(\left[TSC1TSC2\right]+\left[TSC1TSC2pS1387\right]\right)\\ +k_{AA}\left[mTORC1\right]\left(\frac{V_{max}\left(\left[L\right]+C_{LS}\right)}{\left[S\right]+\left[L\right]+C_{LS}}\right)\\ -f_{1}\left(\left[PRAS40\right]\right)-f_{2}\left(\left[ActULK1\right]\right) \end{array}$$

in which [L] denotes leucine concentration, [S] denotes sestrin2 concentration, and C_LS_ denotes the concentration of other aminoacids (e.g., lysine). Besides mTORC1, the developed model additionally simulates the dynamics of NAD+. The model was used to simulate the different dietary conditions and therapeutic treatments. The acute rapamycin treatment was modeled by decreasing total mTORC1 by 75%, while the chronic treatment was simulated by decreasing both mTORC1 and mTORC2 by 75% [100]. The glucose tolerance test was performed by incorporating the equation for change of glucose concentration ([G]) in plasma:

(30) $$\frac{d\left[G\right]}{dt}=k_{G,i}-k_{G,meta}\left[G\right]\left[AKTpT308pS472\right]$$

**Figure 10:**
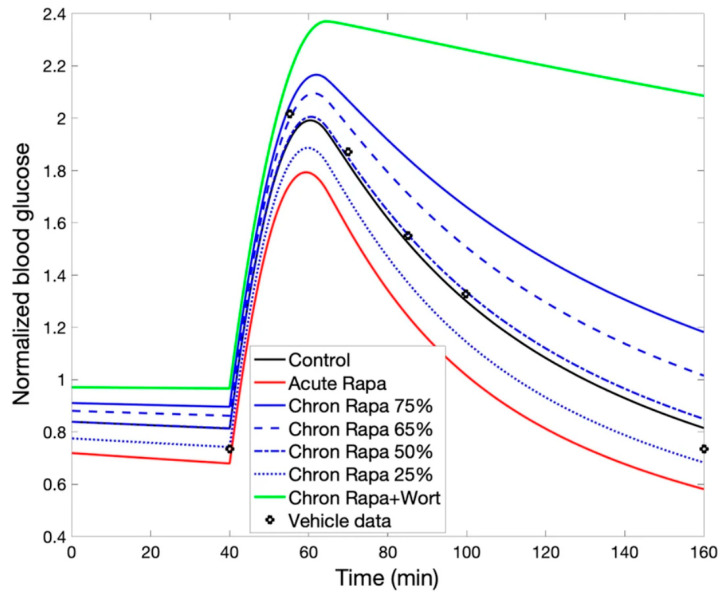
Results of simulated glucose tolerance test for acute and chronic (at various dosage) rapamycin administration. Vehicle data were taken from literature [101]. Reproduced under Creative Commons license from ref. [100] with no changes.

Authors modeled the cellular uptake rate of glucose in plasma, which is a first step in the glucose cellular metabolism, as proportional to the concentration of AKT_pT308_pS472 in a cell [100]. Thanks to their approach, authors were possible to simulate the glucose tolerance test (see Figure 10)—the model was started at the fasting state (low insulin level), assuming [G] = 1 and k_G,i_ = 0 (no glucose intake), while k_G,meta_ was set as 0.0007 min [100]. The test begins at t = 40 min—at this moment k_G,i_ is set to 0.115 min^−1^ and then it linearly decreased to 0 in 25 min [100]. The level of insulin was assumed to be proportional to the glucose concentration in plasma.

**Figure 11:**
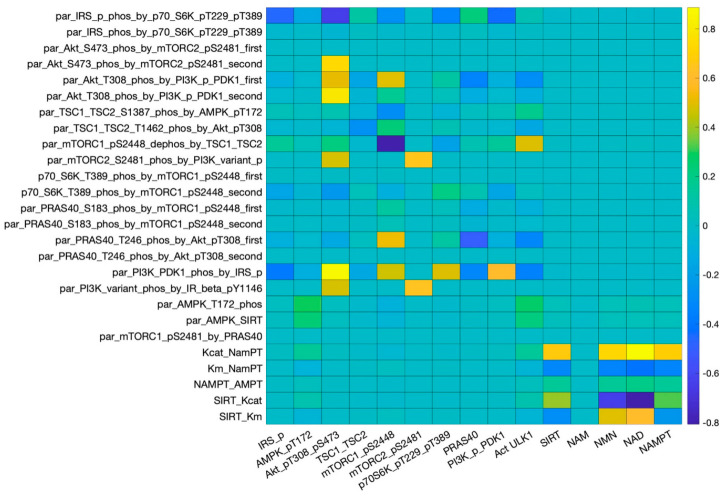
Results of sensitivity tests for key model outputs (presented at horizontal axis) to selected model parameters (presented at vertical axis). Reproduced under Creative Commons license from ref. [100] with no changes.

Authors were also able to perform sensitivity analysis for various parameters (p) included in the model, based on change in model output (x) according to the following equation:

(31) $$Sensitivity=\frac{\left(x\left(p+\Delta p\right)/x\left(p\right)\right)-1}{\Delta p/p}$$

where Δp was set to 0.01p [100]. Thanks to that it was possible to determine the most important parameters in the model (results are presented in Figure 11). This analysis revealed the presence of two distinguishable regions corresponding to the two biggest model components: the Preiss–Handler and salvage signaling pathways (bottom-right region in Figure 11), and the insulin/IGF-1 signaling pathway (upper-left region in Figure 11) [100]. 

Additionally, thanks to the simulation of models, authors have found that the PRAS40 is an important factor in control of tumor growth and that sestrin2 may serve as a therapeutic target for metabolic and neurodegenerative diseases, and cancer [100]. Authors also speculate that the modifications of diet may improve the efficiency of therapies targeting mTORC1 and sestrin2 as the latter’s inhibitory effect on mTORC1 activity is strongly influenced by aminoacids (e.g., leucine) [100]. Lastly, modeling indicated that sufficiently high dosages of STACs may cause premature autophagy, by activating SIRT1 [100]. 

##### **ODE-Based Mechanistic Modeling of AMPK-mTORC Signaling with the Purpose of Unraveling of AMPK Role in Cancer** 

**Figure 12:**
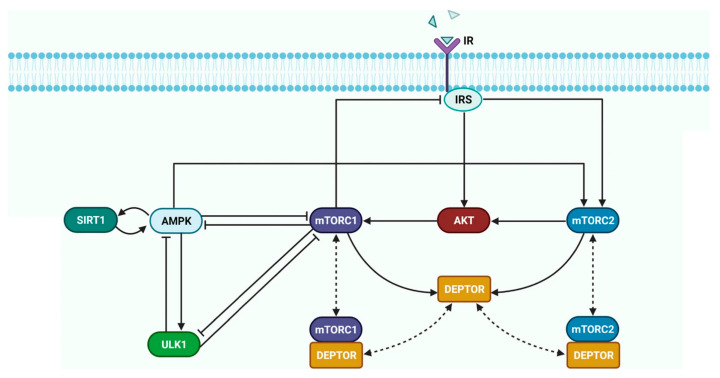
Scheme of model of metabolic pathway used by Sadria and cooperators. Normal arrows denote activation, blunt arrows denote inhibition, and dashed arrows denote formation of complex. Reproduced under Creative Commons license from ref. [102] with no changes.

Cancer cells have specific energetic and synthetic demands so they are characterized by altered nutrient acquisition and metabolism pathways [102]. AMPK (AMP-activated protein kinase) is a major factor that regulates the homeostasis and cellular metabolism in all kind of cells [102]. This kinase has a double-sided role in the development of cancer—initially, it suppresses the growth of tumor, but once it is formed it may promote its growth or further suppress it (depending on circumstances) [103,104,105]. AMPK inhibits mTORC1, an mTOR complex, which supports cell proliferation, and activates mTORC2 [102]. Sadria and cooperators have developed and utilized an ODE-based model of an AMPK-mTORC signaling pathway (Figure 12) to investigate the controversial influence of AMPK on cancer cells [102]. The model was fitted to the available experimental data.

**Figure 13:**
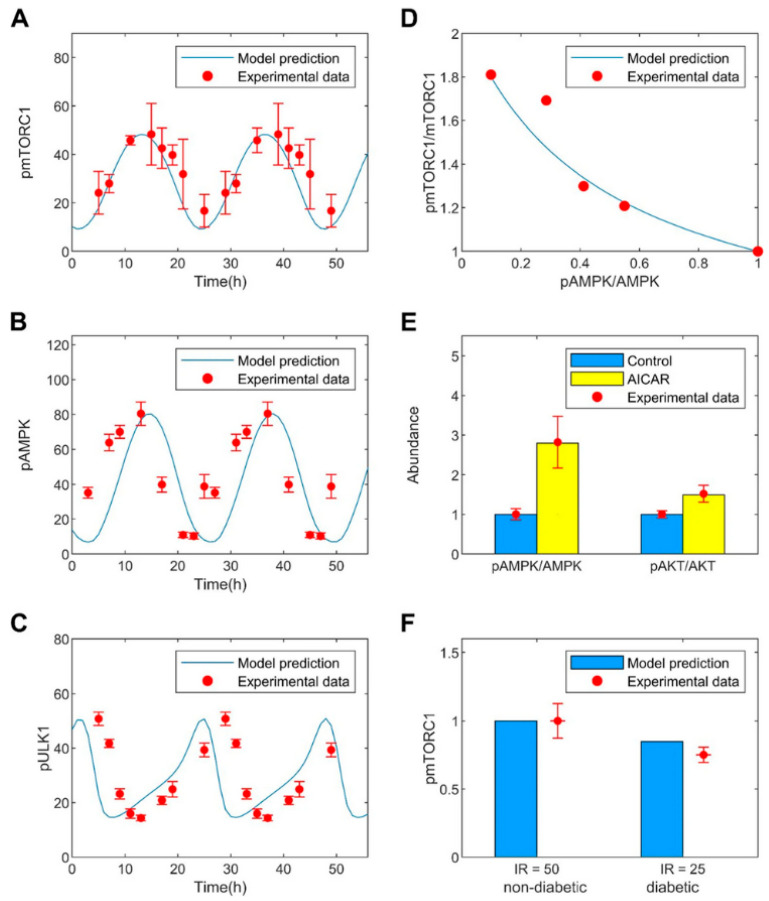
The results of fitting of model used by Sadria and cooperators. (**A**–**C**) represent the oscillatory behavior under energetic stress. (**D**) presents predicted ratio of mTORC1 phosphorylated and unphosphorylated as a function of AMPK activation. (**E**) presents computed activation of AKT and AMPK after administration of AICAR. (**F**) presents modeled abundance of phosphorylated mTORC1 varying level of insulin receptors (IR). Reproduced under Creative Commons license from ref. [102] with no changes.

Model developed by Sadria and cooperators included proteins that are crucial in cellular metabolism: mTORC1, mTORC2, DEPTOR, and ULK1 (the latter mediates signal of mTORC1 during initiation of autphagy), insulin receptor substrate (IRS) and AKT (the latter modulates a vast part of insulin’s metabolic effect), and AMPK and SIRT1 (two main energy sensors) [102]. Although the insulin/mTOR pathway has been previously simulated many times, the research presented by Sadria and cooperators was the first to incorporate DEPTOR, AMPK, SIRT, and ULK simultaneously [102]. Ordinary differential equations, constituting the model, were constructed using the law of mass action and involved the Michaelis–Menten type kinetics [102]. The whole model contained in total 20 ODEs and 58 parameters [102]. The model was calibrated on plausible multiple experimental data so that it meets three criteria (Figure 13) [102]. The first criterion was the ability to predict oscillations in mTORC1, ULK1, and AMPK under the condition of energetic stress (Figure 13A–C) [102]. The second criterion stated that the model should approximate the experimental data of feedback response of mTORC1 varying AMPK (Figure 13D) [102]. The third criterion required reproducing experimental data of AMPK and AKT activity in response to the treatment of AICAR (Figure 13E) [102]. All three criteria were satisfied simultaneously in an iterative way. The first criterion was fulfilled using interior point algorithm implemented in MATLAB fmincon built-in function [102]. The second criterion was fulfilled by matching the values of parameters K_AMPK and K_AMPK_by_SIRT1 so they minimize the least-square difference between mTORC1 and mTORC2 predicted response curves while changing the fractional activation of AMPK in range 0.1–1.0 [102]. The third criterion was met by manipulating the values of K_AMPK and K_AMPK_by_SIRT1 parameters to match the experimental AMPK and AKT activation levels [102]. 

**Figure 14:**
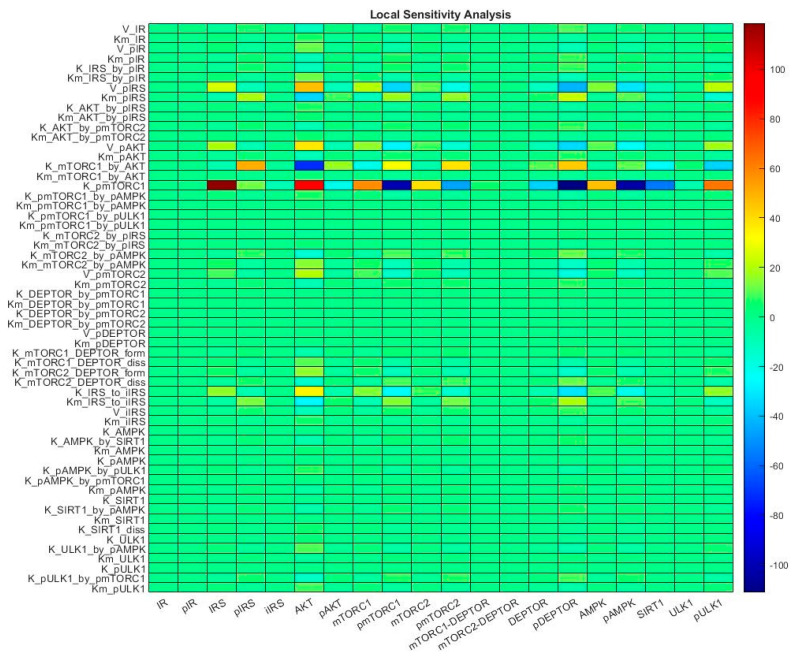
The results of local sensitivity analysis performed by Sadria and coauthors. Horizontal axis—model outputs, vertical axis—model parameters. Reproduced under Creative Commons license from ref. [102] with no changes.

**Figure 15:**
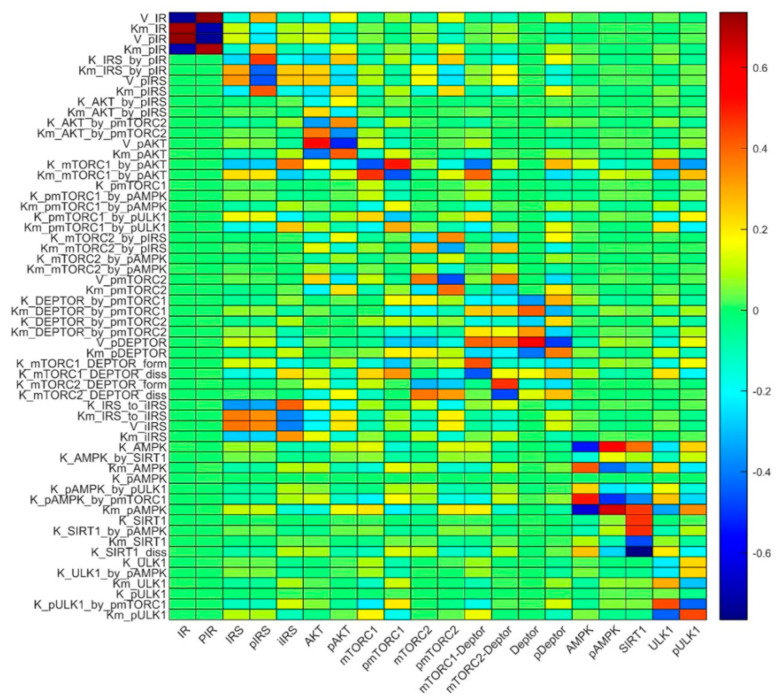
The results of global sensitivity analysis performed by Sadria and coauthors. Horizontal axis—model outputs, vertical axis—model parameters. Reproduced under Creative Commons license from ref. [102] with no changes.

The authors performed bifurcation, local sensitivity, and global sensitivity analyses of the model. The local sensitivity analysis showed how small changes in individual parameters (p) of the model influenced the output signals (S) (Figure 14). It was computed as a normalized derivative of S with respect to the parameter:

(32) $$LocalSensitivity=\frac{dS/S}{dp/p_{0}}\approx \left(\frac{p_{0}}{S\left(p_{0}\right)}\right)\frac{S\left(p_{0}+\Delta p\right)-S\left(p_{0}\right)}{\Delta p}$$

where S(p_0_) denotes the concentration of signal S in the steady-state calculated using initial set of parameters (p_0_). During these tests, the parameters of interest were changed by 0.5% [102]. In contrast, during the global sensitivity analysis the output variables were calculated using parameter values from the whole space defined by the physiological ranges (Figure 15) [102]. Sadria and cooperators have used 100,000 sets of parameter values that were randomly chosen from the whole parameter space with the aid of Latin hypercube sampling [102]. Partial rank correlation coefficients (PRCC), that show the correlation between parameter set (p_0_) and S in steady-state, were calculated using the equation below:

(33) $${PRCC}_{\vec{p_{0}},\vec{S}}=\frac{Cov(\vec{p_{0}},\vec{S})}{\sqrt{Var(\vec{p_{0}})Var(\vec{S})}}$$

Local sensitivity analysis revealed that most of the parameters have a negligible effect on model variables, except the K_pmTORC1 parameter which controls the rate of deactivation of mTORC1. This parameter influenced most of the model outputs that gave evidence for determination of pathway dynamics by mTORC1 [102]. On the other hand, the global sensitivity analysis allowed for the identification of parameters in which manipulating allows for the control of levels of certain proteins [102]. In this particular case the most interesting were the parameters that allow the suppression of proliferation and survival of cancer cells [102]. The global sensitivity analysis showed that there is no such parameter to which both mTORC1 and mTORC2 are highly susceptible [102]. However, this analysis indicated two parameters: the deactivation rate of mTORC2 and the dissociation rate of the mTORC2-DEPTOR complex as the most promising drug targets in cancer therapy [102]. 

Other performed computations predicted that the nutrient availability determines the effect of increasing AMPK activity on mTORC and that indirect inhibition of activity of AMPK (via SIRT1 inhibition) affects mTORC activity at a low availability of nutrients [102]. Moreover, direct inhibition of mTORC2 and simultaneous AMPK activation is a promising strategy for curbing cell proliferation and survival [102]. 

##### **A Model for Identification of Potential Therapies for PIK3CA-Mutant Breast Cancer** 

The identification of the molecular and cellular circumstances that lead to cancer gives promise to the development of effective anti-cancer therapies. PIK3CA is a gene encoding the p110α subunit of PI3K [106]. Its mutation is characteristic for estrogen receptor positive-type breast cancer [106,107]. For that reason PI3K is a target for various proposed drug agents, for example alpelisib (BYL719) which is an PI3Kα inhibitor is approved for treatment of PIK3CA-mutant metastatic breast cancer [108,109]. Nevertheless, the inhibition of PI3Kα may be resistant due to the incorporation of secondary mutations in compensatory genes and does not allow for the achievement of stable tumor remission [107,110,111]. This is a motivation behind searching for co-targets complementing agents targeting only PI3Kα [107].

Yip et al. constructed a mathematical model which represented the PI3K signaling pathway for two cell states: sensitive and resistant to BYL719 [107]. The model incorporated already known mechanisms behind the resistance to BYL719 and was used to study the effect, on cell proliferation, of targeted inhibition of different members of the PI3K signaling network combined with the inhibition of PI3Kα [107]. Authors found that the most promising co-target for PI3Kα is PDK1. Such a combination allows for the suppression of the activation of biomarkers of resistance and the enhancement of response of T47D cells to alpelisib [107]. The model contained 64 reactions, 72 ordinary differential equations for the integrated PI3K signaling pathway and 5 ODEs for the phenotypic model [107]. MATLAB and IQM toolbox were used to perform calculations, together with the SUNDIALS suite (Suite of Nonlinear and Differential/Algebraic equation solvers). The parental PI3K signaling network model was fitted to the experimental data with the aid of genetic algorithms from the MATLAB Global Optimization Toolbox [107]. 

##### **A Dynamic Model of PI3K/mTOR Signaling Pathway Revealing Biphasic Dependence of Activity of mTORC1 on the SIN1 Subunit of mTORC2** 

PI3K/mTOR signaling pathways are responsible for the regulation of many important cellular processes, such as cell metabolism, growth, and autophagy [112,113]. mTOR kinase is a key catalytic subunit of mTORC1 and mTORC2 complexes which have distinct functions [112]. Other common components of these two complexes are DEPTOR and GβL [112]. The action of mTORC1 and mTORC2 is coordinated but still in an unknown way due to, among others, a complex network interaction including many feedback loops and post-translational modifications [112]. Ghomlaghi and cooperators constructed a novel mechanistic model incorporating crucial details such as the AKT-mTORC2 positive feedback loop and coordination of mTORC1 and mTORC2 by MLST8 ubiquitination [112]. The model was simulated and validated based on the experimental data, and it revealed an unknown so far biphasic dependence of mTORC1 activity on the SIN1 subunit of mTORC2 [112]. The analysis performed by Ghomlaghi and coauthors also showed that the ubiquitination of MLST8 is controlled by IRS1 and IRS2, and suggests that MLST8, enabling mTORC1 and mTORC2 activity, may be a potential target in breast cancer therapy [112]. 

**Figure 16:**
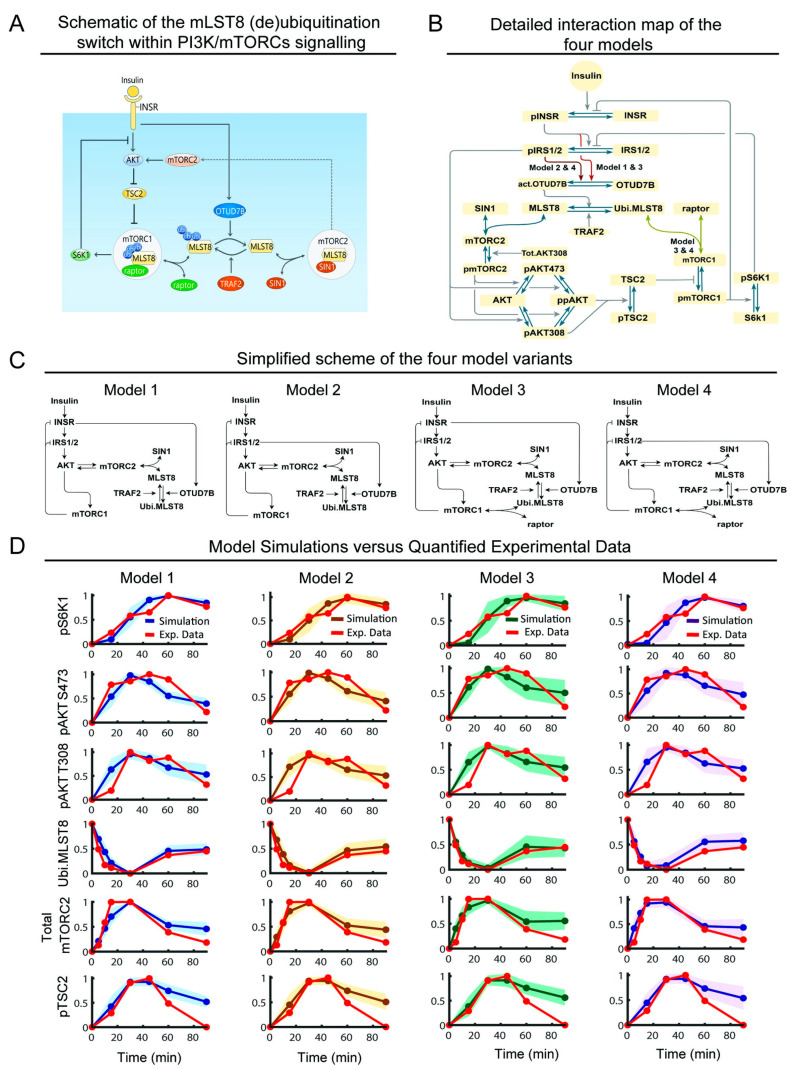
Scheme of modeling of the PI3K/mTOR signaling pathway performed by Ghomlaghi and cooperators. Reproduced under Creative Commons license from ref. [112] with no changes.

**Figure 17:**
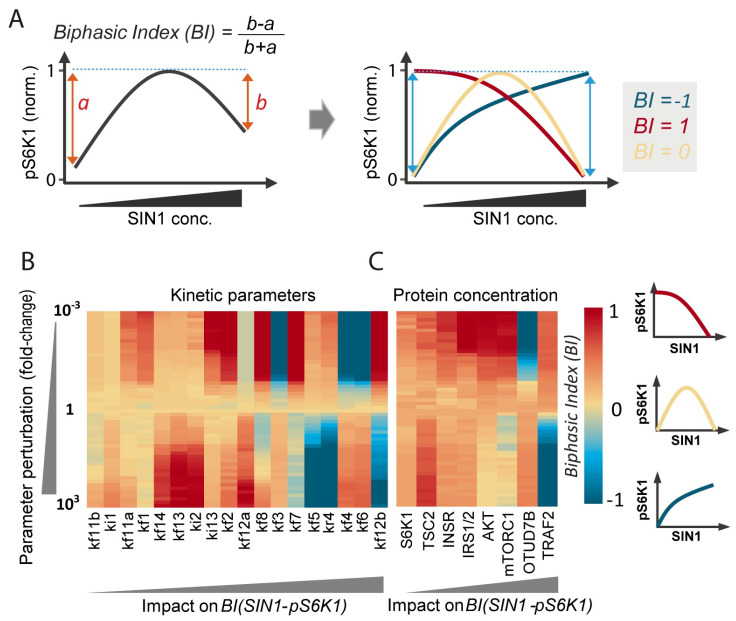
Chart presenting sensitivity analysis performed by Ghomlaghi and cooperators: (**A**) definition of the biphasic index, (**B**) impact of model kinetic parameters perturbation on the BI, (**C**) impact of the protein concentrations perturbation on the BI. Reproduced under Creative Commons license from ref. [112] with no changes.

The model proposed by Ghomlaghi et al. were the first to explicitly involve the regulation of formation of mTORC1 and mTORC2 complexes by MLST8 (de)ubiquitination switch [112,114]. The implemented signaling pathways are presented in Figure 16A. The way in which the MLST8 (de)ubiquitination switch interacts with other mechanisms of regulation present in the PI3K/mTOR signaling network was unclear so authors proposed new models which are presented as model reaction scheme in Figure 16B [112]. It includes crucial feedback and signaling events started by the insulin stimulation. To fill the gaps in an adopted signaling network, authors considered four different variants of the model (Figure 16C) [112]. Using these models authors checked if the activity of OTUD7B is regulated by the S6K1 negative feedback and if the mTORC1 kinase activity requires MLST8 [112]. All of the variants were expressed in terms of ordinary differential equations. By solving the system of model ODEs authors were able to predict the temporal evolution of protein concentrations in the network. The rates of protein–protein interactions were expressed using the law of mass action while the rates of enzymatic reactions (such as the (de)ubiquitination) or (de)phosphorylation) were described by kinetics of Michaelis–Menten type [112]. The model included 22 ODEs in total and 41 (for variants 1 and 2) or 43 (for variants 3 and 4) parameters [112]. Authors estimated parameters of model using literature experimental data from mouse embryonic fibroblast cells [112]. For that purpose authors utilized an genetic algorithm implemented in MATLAB software (specifically, the Global Optimization Toolbox along with the ga function) [112]. The fitting procedure was repeated independently 500 times for each model. It allowed for determination of as many sets of best-fitted parameters as possible [112]. Ghomlaghi and coauthors also performed a sensitivity analysis to study the molecular factors governing the biphasic behavior of concentration of SIN1 and mTORC1 activity [112]. Such a strategy is less demanding than corresponding experimental studies. For this purpose a biphasic index (BI) was defined that expressed the biphasicness of the pS6K1-SIN1 concentration response curve (Figure 17A) [112]. The concentration of SIN1 was changed from 0.01 to 1000 times its initial value and the pS6K1 was determined. Then each kinetic parameter of the model was varied in range from 0.001 to 1000 of its nominal value and each time the BI was calculated (Figure 17B) [112]. 

The results of sensitivity analysis revealed that the shape of the SIN1-pS6K1 response curve is impacted mostly by the parameters linked with the MLST8 switch, namely the (de)ubiquitination of MLST8, OTUD7B activation, and the MLST8-SIN1 binding [112]. Similar sensitivity analysis performed for protein concentrations (Figure 17C) shows that the perturbation of the primary MLST8 (de)ubiquitination switch regulators, TRAF2 and OTUD7B, has the strongest influence on the biphasic index of the SIN1-pS6K1 response curve [112].

#### 2.1.5. Summary

**Figure 18:**
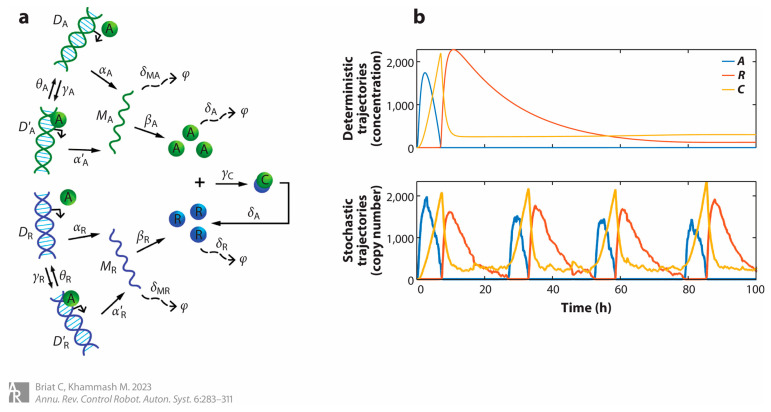
Schematic representation of oscillations generated by molecular noise: (**a**) a model of circadian clock by Vilar and cooperators [118], (**b**) deterministic (**top**) and stochastic (**bottom**) trajectories of repressor molecule (R). A—active molecule, C—complex molecule. Reproduced under Creative Commons license from ref. [116] with no changes.

ODE-based models are easy to construct and implement, and may give insights into the mechanisms of enzymatic reactions or utilize their scaling up. However, the kinetic parameters are generally not known and the model must be fitted to experimental data to obtain values of these parameters. The collection of necessary data may be time- and/or cost-consuming. Another disadvantage of ODE-based models lies in the assumption of homogeneity—these models do not include important factors such as population/concentration heterogeneity and diffusion. The deterministic nature of ODE-based models is also a disadvantage in the context of modeling of relatively small systems, where noise starts playing an important role. In this context stochastic simulations are very important and allow insights into the probabilistic nature of signals emerging from the single-cell analyses in which the signal is strongly affected by molecular noise (Figure 18) [115,116,117,118]. For stochastic simulations a Gillespie algorithm and Monte Carlo methods are widely used [119,120]. Stochastic models are especially useful in investigations of processes like cell fate determination for which the response of an individual cell may differ soundly despite similar conditions [121,122].

## 3. Machine Learning and Artificial Intelligence

The machine learning (ML) and artificial intelligence techniques gained significant attention in the scientific community dealing with the prediction of patterns of pathway activations caused by, for example, genomic mutations and drug acquisition [123,124,125]. ML models (e.g., artificial neural networks (ANN) and support vector machines (SVMs)) require prior training on experimental data. Then they may be applied to identify potential drug targets or key regulators in signaling pathways [126,127]. Deep learning algorithms may simulate the outcomes of pharmacological and genetic perturbations in signaling pathways with high efficiency and accuracy. As an example, recurrent neural networks (RNNs) are capable of prediction of temporal patterns in signaling and gene regulatory networks providing an efficient alternative for ordinary differential equations [128,129]. On the other hand, convolutional neural networks are used for analysis of images of cells allowing for the mapping of spatial dynamics of signals [130,131].

**Figure 19:**
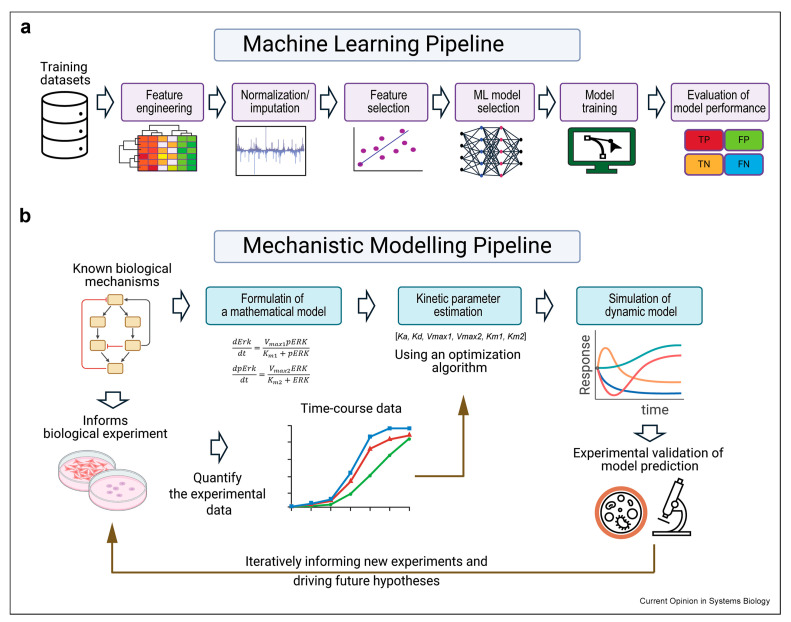
A comparison of generalized workflows for machine learning (ML)-based modeling and ordinary differential equation (ODE)-based modeling. Reproduced under Creative Commons license from ref. [132] with no changes.

Although the mechanistic-based models utilizing ordinary differential equations are easily explainable, they scale poorly with the system size that makes data acquisition demanding and limits their applications [132]. Machine learning models are generally more cost-effective in terms of computation; however, their interpretation is often difficult [132]. Nevertheless, recent efforts eased the interpretation by the development of algorithms such us LIME, SHAPLEY, and permutation importance algorithm [133,134,135,136,137]. Figure 19 depicts a comparison between a typical workflow for ML-based and ODE-based modeling outlining typical differences between these two approaches.

### 3.1. Examples of Applications of ML-Based Models for Modeling of Biochemical Reactions and Cell Signaling

#### 3.1.1. ANN-Based Intelligent Framework for Modeling of Nonlinear Irreversible Biochemical Reactions

Irreversible biochemical reactions are crucial for the dynamics of cellular processes. Catalyzed by enzymes, biochemical reactions often exhibit nonlinear behavior that makes their modeling with ordinary differential equations harder. Bilal and cooperators proposed a computational framework based on artificial neural networks for modeling of the nonlinear irreversible biochemical reactions (NIBRs) [138]. The authors considered reactions exhibiting extended Michaelis–Menten kinetics, that is taking into account not only the enzyme–substrate complex, but also the enzyme–product complex [138]:

(34) $$S+E\begin{array}{c} \overset{k_{1}}{\to }\\ \underset{k_{-1}}{\leftarrow } \end{array}ES\begin{array}{c} \overset{k_{2}}{\to }\\ \underset{k_{-2}}{\leftarrow } \end{array}EP\overset{k_{3}}{\to }E+P$$

Such kinetics included five distinct kinetic parameters involved in the following system of ODEs:

(35) $$\frac{d\left[S\right]}{dt}=-k_{1}\left[E\right]\left[S\right]+k_{-1}\left[ES\right]$$

(36) $$\frac{d\left[ES\right]}{dt}=k_{1}\left[E\right]\left[S\right]-k_{-1}\left[ES\right]-k_{2}\left[ES\right]+k_{-2}\left[EP\right]$$

(37) $$\frac{d\left[EP\right]}{dt}=k_{2}\left[ES\right]-k_{-2}\left[EP\right]-k_{3}\left[EP\right]$$

(38) $$\frac{d\left[P\right]}{dt}=k_{3}\left[EP\right]$$

Datasets for training and validation of the model (a multilayer feedforward artificial neural network) were generated by numerical integration of relevant ODEs employing the fourth order Runge–Kutta algorithm with the following initial conditions [138]:

(39) $$\left[S\right]\left(0\right)={\left[S\right]}_{0}$$

(40) $$\left[ES\right]\left(0\right)=\left[EP\right]\left(0\right)=\left[P\right]\left(0\right)=0$$

**Figure 20:**
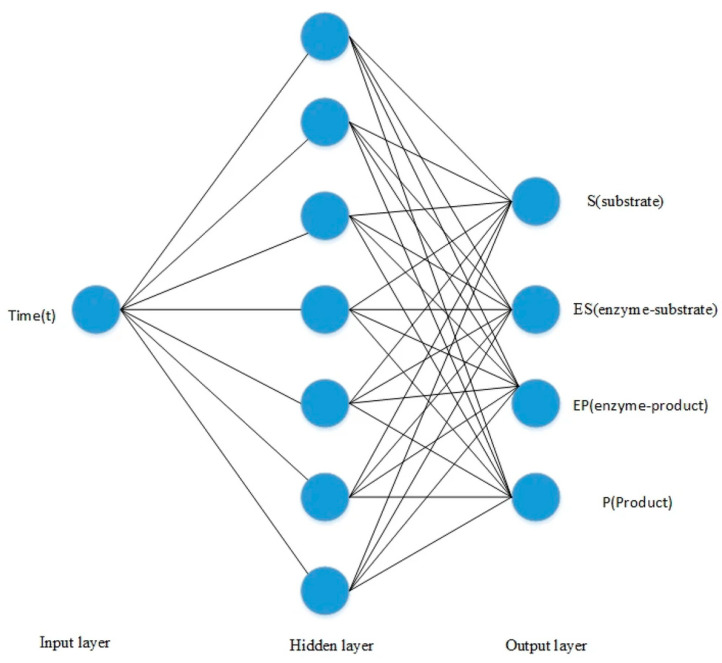
A topology of multilayer perceptron used in the Bilal et al. study [138]. Reproduced under Creative Commons license from ref. [138] with no changes.

**Figure 21:**
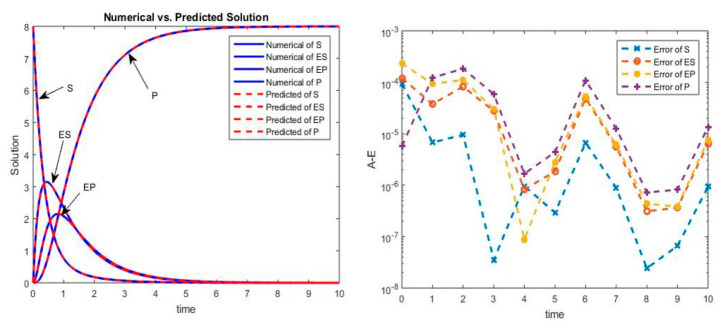
A sample plot comparing numerical and ANN predicted values in Bilal et al. study [138]. Reproduced under Creative Commons license from ref. [138] with no changes.

The training of the neural network was performed using the backpropagation Levenberg–Marquardt (BLM) algorithm [138]. For that reason authors refer to their main model as BLM-ANN, in contrast to another two studied algorithms, Scaled Conjugate Gradient (SCG) and Bayesian Regularization (BR) [138]. Input for ML models was time point and outputs were the concentrations of reagents (substrate, enzyme–substrate complex, enzyme–product complex, product) at this time point. The ANN-based model, which utilized multi-layer perceptron (MLP) architecture (Figure 20), outperformed BR and SCG models regarding prediction accuracy (which for BLM-ANN model achieved values of order of magnitude 10^−13^ in terms of mean-squared error), convergence speed, and validity for various kinetic characteristics (see Figure 21 as an example).

#### 3.1.2. The Oscillations in Dynamics of mRNA Metabolism and Chromatin Accessibility During Cell Cycle Revealed by Multiomics and Deep Learning

**Figure 22:**
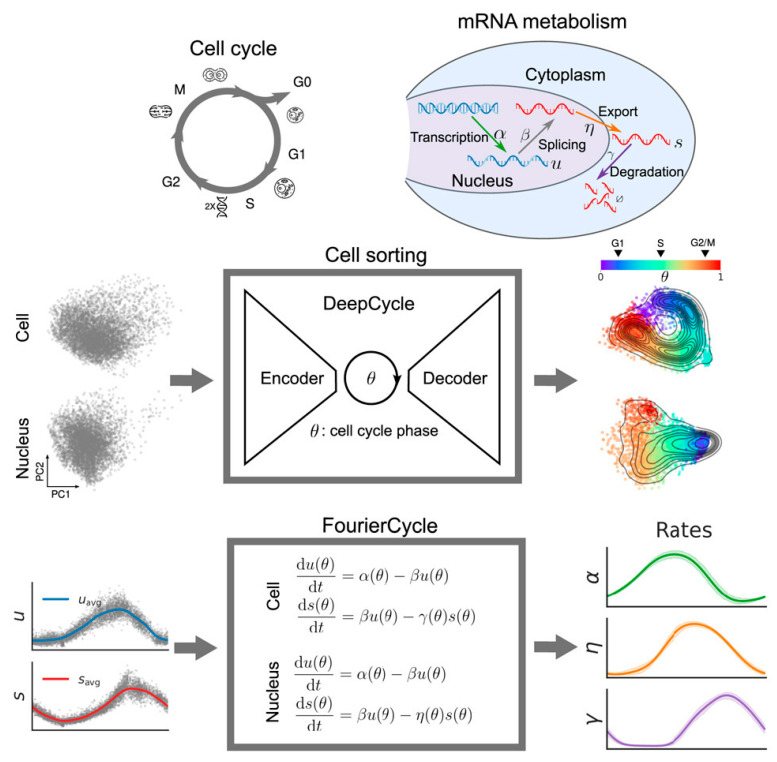
A schematic presentation of investigation performed by Nariya et al. [139]. Reproduced under Creative Commons license from ref. [139] with no changes.

A precise temporal expression of thousands of genes is necessary for stabilized cell cycle [139]. Nariya and cooperators utilized multiome sequencing of single-cell, biophysical modeling, and deep learning to determine the rates of degradation, nuclear export, splicing, and transcription of mRNA [139]. Their study discovered oscillatory waves in transcriptional and post-transcriptional processes at specific phases of cell cycle. Thanks to adopted computational approach researchers were also able to elucidate the key factors underlying the oscillatory mRNA dynamics [139]. In their past study the authors developed a deep learning tool, called DeepCycle, that uses single-cell RNA sequencing (scRNA-seq) with the aim of mapping gene expression profiles to a variable describing the phase of the cell cycle (denoted as Θ) [139,140]. They used the DeepCycle approach to investigate the dynamics of expression of genes in somatic and embryonic cells discovering waves of RNA accumulation in the course of the cell cycle [139,140]. Their findings opened a new question about the way in which the processes governing the RNA accumulation are temporally regulated on a genome-wide scale in the course of the cell cycle [139,140]. Researchers addressed this question using a combination of DeepCycle, single-cell RNA sequencing, single-nucleus multiome sequencing, and construction of biophysical model, calling their approach FourierCycle [139]. Thanks to it they were able to identify key transcription factors together with the dynamics of their binding activities in the course of the cell cycle via examination of changes the accessibility footprints of chromatin near their binding sites [139]. Figure 22 presents a summary of study performed by Nariya et al. [139].

#### 3.1.3. Machine Learning-Based Risk Model for Glioma

Glioma is a major type of brain tumor with increasing incidence over several past years [141,142]. A precise prognosis and early diagnosis are crucial for its treatment. Glioma is possibly affected by the MAPK (mitogen-activated protein kinase) signaling pathway with unexplored clinical values [143]. Liu and Tang addressed this issue by identifying up-regulated MAPK signaling pathway genes in glioma and using them to cluster subtypes of glioma by utilizing consensus clustering. They also trained a prognostic model using the identified genes and machine learning model, specifically the least absolute shrinkage and selection operator (LASSO) regression algorithm [143]. LASSO is capable of performing both regularization—with the aim to increase the accuracy of prediction and interpretability of the model—and variable selection [143]. LASSO selected the gene signatures and constituted a model for prognosis of overall survival [143]. The authors analyzed the correlations between cancer-associated signatures in cancer and their risk model [143]. Hub gene and survival analyses were utilized to identify the crucial hub genes of the gene set [143]. It turned out that 47% of genes of the MAPK signaling pathway were overexpressed in glioma [143]. Liu and Tang developed a risk model of high confidence for the prediction of total survival and correlated it with cancer-associated signatures. From all identified 12 hub genes eight were linked with survival. The construction of the prognostic model was performed based on the “glmnet” R package [143,144].

#### 3.1.4. Machine Learning- and Deep Learning-Based Prediction of Turnover Numbers of Enzymes

**Figure 23:**
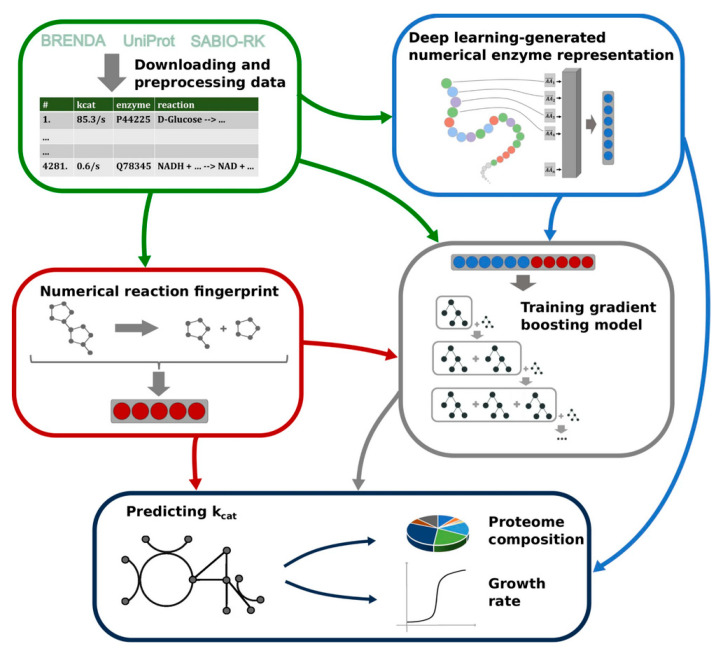
A schematic presentation of workflow in the Kroll et al. investigation [145]. Reproduced under Creative Commons license from ref. [145] with no changes.

The turnover number (k_cat_) characterizes enzyme efficiency. Its experimental determinations for many enzymatic reactions are unavailable [145]. For that reason it is important to develop computational models capable of prediction of turnover number [145]. Machine learning models constructed before the Kroll et al. study were inefficient or limited to single organism [145]. For that reason Kroll and cooperators developed TurNuP, a new machine learning model that was organism-independent and capable of successful prediction of turnover number for natural reactions involving wild-type enzymes [145]. The representation of chemical reactions, constituting inputs for the model, were made using differential reaction fingerprints [145]. The representation of enzyme also constituted the model input and was realized by a Transformer Network model (a natural language processing model) for protein sequences [145]. Enzyme and reaction representations were concatenated before being used as training data for a gradient boosting model [145]. The model developed by Kroll and coauthors (Figure 23) was more accurate than the previous ones. The authors also constructed a TurNuP web server to share their tool for biochemical and physiology studies [145]. Values of turnover number predicted with TurNuP were used to parametrize metabolic models that led to improved predictions of proteome allocation [145].

Kroll et al. not only used the gradient boosting algorithm, but for comparison also used a fully connected artificial neural network model (from TensorFlow library), random forest model, and linear regression model (the latter two from scikit-learn Python package) [145]. 

#### 3.1.5. Improved Kinetic Modeling Through Machine Learning-Aided Global Optimization for Determination of Michaelis Kinetic Constant

**Figure 24:**
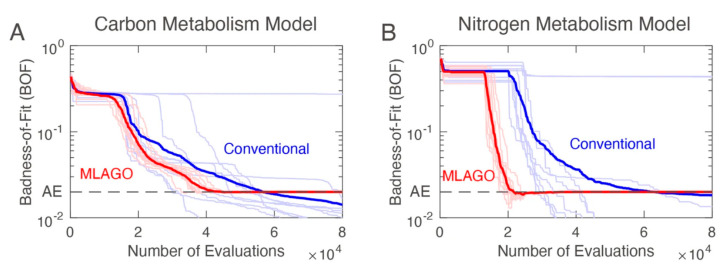
Comparison of performance of conventional method (blue) and the MLAGO method (red) for carbon (**A**) and nitrogen (**B**) metabolism models. The dashed black lines represent the allowable error (AE). Reproduced under Creative Commons license from ref. [146] with no changes.

The dynamic behavior of biochemical systems may be studied with kinetic models which require determination of values of kinetic parameters, e.g., the Michaelis constant [146]. For the purpose of parameter estimation global optimization techniques have long been used. However, these are facing three main problems: relatively high computational costs, possibility to fall into suboptimal local minimum, and difficulties in identification of a unique solution [146,147]. Maeda et al. addressed these issues by proposing a novel approach, the Machine Learning-Aided Global Optimization (MLAGO), for determination of the Michaelis constant [146]. The approach constitutes two steps. Firstly, the Michaelis constant is predicted using machine learning based on three factors: organism ID, KEGG compound ID, and EC number [146]. The authors used a well-curated database of Michaelis constant provided by Bar-Even et al. [146,148]. The final dataset used by the authors contained 17,151 values, for 2588 unique EC numbers, 1612 unique compound IDs, and 2212 unique organism IDs [146]. Secondly, the constrained global optimization is performed using such predicted Michaelis constant values as references [146]. Initially five different machine learning algorithms were used: k-nearest neighbors, random forest model, elastic net, TabNet, and the gradient boosting model, from which the random forest model achieved the best scoring and was chosen for further analyses [146]. The MLAGO approach turned out to be more efficient and less computationally expensive than conventional methods according to benchmark tests performed on the carbon and nitrogen metabolism models (Figure 24) [146,149,150].

#### 3.1.6. AlphaFold—Deep Machine Learning Framework for Prediction of Protein Structure

**Figure 25:**
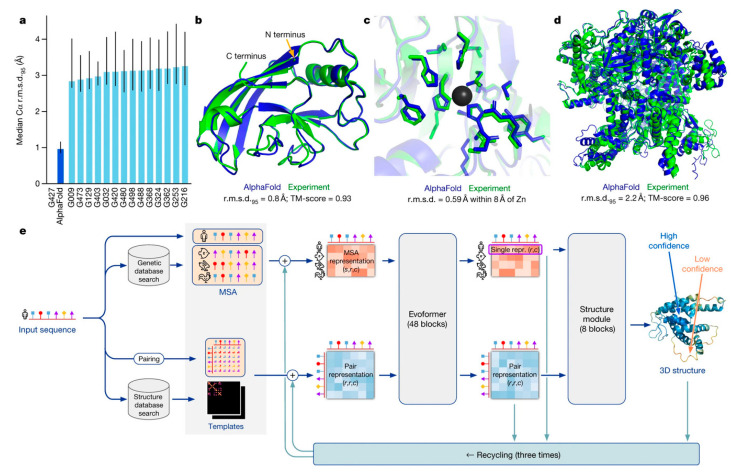
A graphical summary of AlphaFold machine learning framework: (**a**) a comparison of different models evaluated on the CASP14 dataset; (**b**) a comparison of experimental (green) and predicted by AlphaFold (blue) structure of sample protein (PDB 6Y4F); (**c**) prediction of zinc-binding site by AlphaFold (PDB 6YJ1); (**d**) correct domain packing of structure predicted using AlphaFold containing 2180 residues (PDB 6VR4); (**e**) the architecture of AlphaFold framework (s—number of sequences, r—number of residues, c—number of channels). Reproduced under Creative Commons license from ref. [151] with no changes.

AlphaFold is a quite recent machine learning model developed by DeepMind, capable of prediction of a 3D protein’s structure based on its amino acid sequence [151,152,153]. Typical experimental techniques used for such purpose, such as spectroscopic methods, cryo-EM and time-resolved X-ray crystallography, are time- and cost-consuming, and face technical problems [153]. It is thus very convenient to have a computational framework capable of protein structure prediction that does not depend on random sampling and coarse-grained representations like the Monte Carlo and molecular dynamics simulations [153]. AlphaFold proved its advantage over such methods by successfully predicting the structures of c.a. 98.5% of proteins present in the human proteome, including intrinsically disordered proteins and regions, in the 14th Critical Assessment of protein Structure Prediction (CASP14) (see Figure 25a) [151,152,154]. 

The AlphaFold framework as an inputs uses primary amino acid sequence and aligned sequences of homologues and predicts the 3D coordinates for all heavy atoms within a concerned protein [151]. The first stage of the framework uses Evoformer (a neural network block) to process the input data and generate two arrays representing processed MSA (multi-sequence alignment) and residue pairs (Figure 25e). In the Evoformer block a structural hypothesis is proposed and is continuously refined. It is then passed to the structure module that presents an explicit 3D structure of protein (Figure 25e). The AlphaFold framework may be trained on PDB data using supervised learning [151]. Depending on the length of the protein sequence, AlphaFold may predict the structure within graphics processing unit (GPU) minutes or hours [151]. It was used to predict the structure of almost the entire human proteome (that is, 98.5% of human proteins [154]).

#### 3.1.7. Physiological Foundation Modeling for the Screening of Candidates for the Clinical Studies of Metabolic Dysfunction-Associated Steatotic Liver Disease

Metabolic dysfunction-associated steatotic liver disease (MASLD; formerly known as non-alcoholic fatty liver disease, NAFLD) is an illness that touches many people without giving pronounced symptoms [155]. Clinical studies of this disease require adequate participants but performing imaging investigations makes screening of potential candidates hard [155]. Yuan and cooperators developed “Bioprofiles”—a tool based on artificial intelligence that makes patient screening easier [155]. It uses information gathered in routine health records (common lab tests, medical history) to learn physiology patterns [155]. “Bioprofiles” was trained on large datasets (1 million of subjects from the UK Biobank and other sources) and used to evaluate the severity of MASLD without imaging for 45,484 subjects at Vanderbilt University Medical Center [155]. The predictions of the model were in close agreement with the measurements of liver fat performed using magnetic resonance imaging (Spearman coefficient of 0.65 against proton density fat fraction) [155]. The application of this AI-based tool allowed for the significant decrease in people requiring screening by about half [155].

#### 3.1.8. Graph Neural Networks (GNNs) for Metabolic Flux Estimation in Microbial Communities

Many objects and phenomena may be represented in the form of a graph, for example a graph of biochemical reactions [156]. Graphs constitute a mathematical representation of data and may be used to train machine learning models, such as graph neural networks (GNNs) [156,157]. GNNs, first introduced in 2009 by Scarselli and cooperators, are capable of direct processing of most of the graph types such as directed, undirected, cyclic, and acyclic [157]. They were successfully used in practical, real-life fields like drug discovery or proposing new molecules [156]. Mohammad et al. used graph neural networks to predict high-resolution metabolic flux within heterogeneous microbial communities [158]. For that purpose, they used spatial omic data (spatially resolved transcriptomic, proteomic, and metabolomic signals) and interaction graphs [158]. Multi-layer graphs were constructed, in which nodes denote microbial taxa or single cells, while edges denote strength of biochemical interaction and spatial closeness [158]. The GNN was also coupled with a physics-informed module, constructed using rules based on microbial metabolic models, restricting stoichiometry and thermodynamics of model propositions [158]. The constructed model outperformed the classical regression and non-spatial machine learning models in terms of predictive accuracy, computational latency, and convergence during training [158]. The GNN-based model may be used for microbiome engineering, optimization of environmental bioprocesses, and synthetic ecology design [158].

#### 3.1.9. Physics-Informed Neural Networks for Modeling of the Growth of Tumor Cells

Physics-informed machine learning (PIML) is an approach for improved computational modeling of complex phenomena by merging classical black-box machine learning methods with parameterized physical laws [159,160]. Among PIML one may distinguish physics-informed neural networks (PINN), proposed in 2017, which are capable of solving nonlinear problems (e.g., nonlinear diffusivity) often arising in mathematical modeling of biological and biomedical phenomena [159,160]. Although machine learning methods, including deep neural networks, are alone capable of tackling nonlinearity, the incorporation of information from physical laws to machine learning may be beneficial in terms of decreasing the required amount of data as evidenced from examples from computational fluid dynamics [160,161,162]. PINN not only learns from the training data but also obeys constraints derived from physical laws included in loss function [160,162]. On the other hand, inverse problems physical laws are used to constrain the domain [160,162].

A practical application of physics-informed neural networks in biomathematics was, for example, demonstrated by Rodrigues who modeled tumor cell growth [163]. The understanding of this phenomenon is very important in terms of developing effective tumor treatment strategies [163]. Rodrigues used two distinct models of tumor growth: the Verhulst logistic growth model (Equation (41)) and the Montroll power-law model (Equation (42)) with the aim to compare their applicability and advantages over each other [163].

(41) $$\frac{dp}{dt}=kp\left(1-\frac{p}{C}\right)$$

(42) $$\frac{dp}{dt}=kp\left(1-{\left(\frac{p}{C}\right)}^{\Theta }\right)$$

where p is a function of time and denotes population size, t is time, k denotes the growth rate, C is the carrying capacity, and Θ is a parameter indicating the position of the inflection point of the tumor growth curve [163].

**Figure 26:**
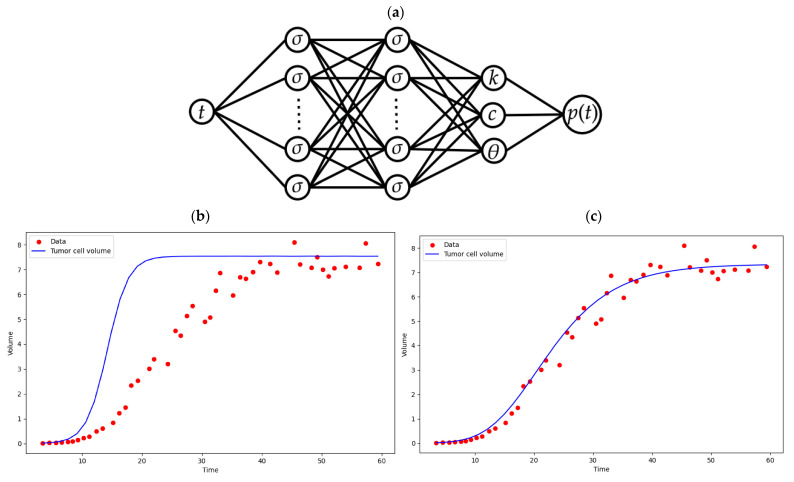
Summary of PINN-based tumor growth modeling performed by Rodrigues: (**a**) architecture of utilized neural network, (**b**) the PINN prediction for the Verhulst model, (**c**) the PINN prediction for the Montroll model. Reproduced under Creative Commons license from ref. [163] with no changes.

Rodrigues used a feedforward multilayer perceptron with the aim to approximate the solutions of the abovementioned growth models (Figure 26a). The input layer represented time while the output layer represented population size. Several hidden layers were included and neurons inside them used various activation functions: sigmoidal function, rectified linear function (so-called rectified linear unit, ReLU), and hyperbolic tangent function [163]. Neurons in the penultimate layer represented the parameters of the growth model (e.g., k, C, and Θ for the Montroll model). The network was trained based on loss function (L_PINN_, Equation (43)) measuring the difference between predicted and experimental data (measured as the mean squared error, L_data_, Equation (44)), but as a physics-informed model, the loss function also included the physics-informed correction (L_physics_, Equation (45)) [163]:

(43) $$L_{PINN}=L_{data}+\lambda L_{physics}$$

(44) $$L_{data}=\frac{1}{N}\cdot \sum_{i=1}^{N}{\left(p\left(t_{i}\right)-y_{i}\right)}^{2}$$

(45) $$L_{data}=\frac{1}{N}\cdot \sum_{i=1}^{N}{\left(p\left(t_{i}\right)-y_{i}\right)}^{2}$$

where λ denotes the regularization parameter, N stands for a number of experimental points, N_R_ stands for a number of points at which the physics-informed restrictions need to be satisfied, and R(t_j_) denotes the residual of the model at the *j*-th point (Equation (46)) [163]:

(46) $$R\left(t_{j}\right)=\frac{d\hat{p_{j}}}{dt}-k\cdot \hat{p_{j}}\cdot \left(1-\frac{\hat{p_{j}}}{C}\right)$$

where p_j_ with the caret symbol is the predicted population size and j ∈ {1, …, N_R_}.

The selection of the value of the regularization parameter λ is very important in terms of convergence of the neural network during training. For two most common types of regularization (Lasso and Ridge types) the value of λ ranges from 0 to positive infinity [163]. The author used data for Chinese hamster V79 fibroblast tumor cell growth taken from the literature [163,164]. After 5000 epochs of training, the Montroll model was better fitted to the experimental data than the Verhulst model (Figure 26b,c) [163].

#### 3.1.10. Machine Learning at the Single-Cell Level—Identification of Tumor Cells

**Figure 27:**
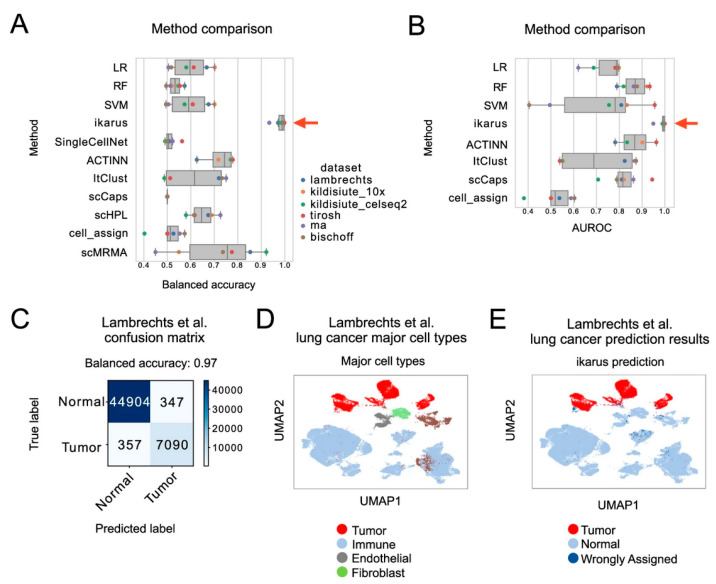
Summary of ikarus machine learning pipeline proposed by Dohmen et al. [167]: (**A**) balanced accuracies for classification of tumor and normal cell for different test datasets and distinct methods, including ikarus; (**B**) AUROC (area under receiver operating characteristic) performance for distinct methods of classification; (**C**) ikarus performance evidenced by confusion matrix on the example of Lambrechts lung cancer dataset; (**D**) experimental cell type indication for the Lamberchts lung cancer dataset; (**E**) cell type indication proposed by ikarus. Reproduced under Creative Commons license from ref. [167] with no changes.

Machine learning may also be applied to study phenomena at the single-cell level, for example to better understand the causative role of genes in complex biological processes or to support transcriptome profiling of cells [165,166]. This is especially important in the context of cancer therapy which often demands precision and personalized medicine due to the variability of the process of tumor growth [167]. Dohmen and cooperators utilized machine learning with the purpose of distinguishing tumor cells from normal cells at the single-cell level based on single-cell sequencing within the tumor [167]. They developed and tested a machine learning pipeline, called ikarus [167]. It acts in two steps: first, it discovers (based on analysis of annotated single-cell datasets) the gene set which constitutes a tumor cell signature; second, it trains a classifier based on the logistic regression algorithm to distinguish between normal and tumor cells [167]. As the gene data may vary with the applied sequencing technique, as well as with the source of cells, authors tested their machine learning pipeline on many different single-cell datasets of various cancer types and obtained with various sequencing technologies [167]. The authors also applied copy number variations, obtained using inferCNV, to improve the classification results [167,168]. The combined approach allowed for the achievement of relatively high sensitivity and specificity (see Figure 27) [167].

#### 3.1.11. Large Language Model for Evaluation of Reliability of Cell Type Annotation

**Figure 28:**
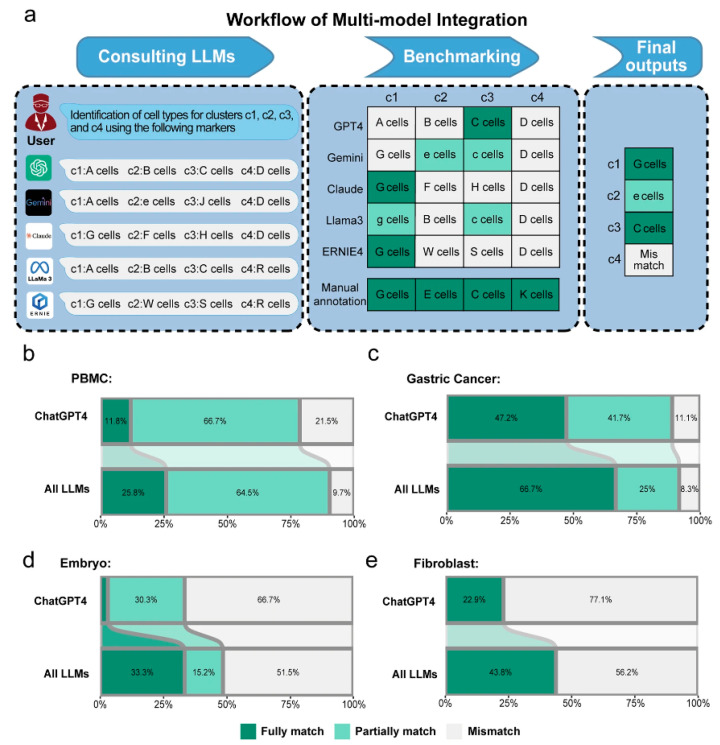
Graphical representation of the LICT: (**a**) workflow for the multi-model integration strategy; (**b**–**e**) bar plots showing comparison between performance of ChatGPT-4 and the integrated set of five LLMs for four different datasets. Reproduced under Creative Commons license from ref. [174] with no changes.

Large language models (LLMs) improved analysis and processing of complex biological data showing potential for bioinformatic studies [169,170]. Applications of LLMs in biomathematics concern the prediction of structure of large molecules like nucleic acids and proteins, text mining of scientific literature, drug design, disease diagnosis, vaccine development and omics [169,170]. LLMs are a part of natural language processing (NLP) and are based on deep neural networks [169,170]. They are capable of interpreting complex data patterns, modeling context, and generating human-like communications [169,170]. Their applicability in bioinformatics relies partially on the apparent similarity between biological sequences and human language, as traditionally language models were desired to process and generate human-like texts [169,170]. For successful operation LLMs require high-quality large-scale training data used in unsupervised pre-training and subsequent fine-tuning [169,170]. A certain example of LLM applied in bioinformatics (specifically: in cell annotation and interpretation of the single-cell sequencing data) is GPTCelltype which takes advantage of the ChatGPT (GPT-4) model and classifies cells with high accuracy [171]. There are more examples of LLM application in the analysis of single-cell sequencing data and in single-cell biology [172,173,174]. For example, Ye and cooperators developed LICT—a Large Language Model-based Identifier for Cell Types—which was validated with various datasets and exhibited better accuracy and reliability than already existing tools for single-cell RNA sequencing analysis [174]. This was done by in fact merging five of the best LLMs found in the literature into one combined system, outperforming each standalone component (see Figure 28) [174]. The strategy for integration was the following: first, five distinct LLMs were prompted to generate their predictions on cell types, then the predictions were compared with manual annotations, and finally the annotation with the highest accuracy among five LLMs was chosen and presented as the final output [174]. 

#### 3.1.12. Summary of ML-Based Models

**Figure 29:**
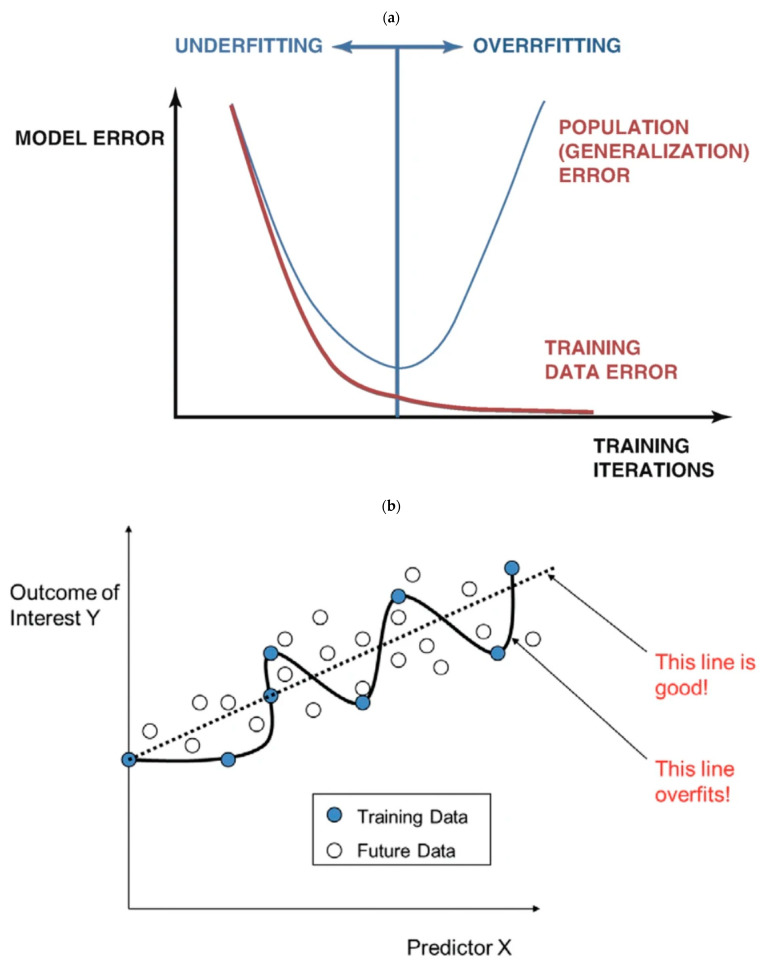

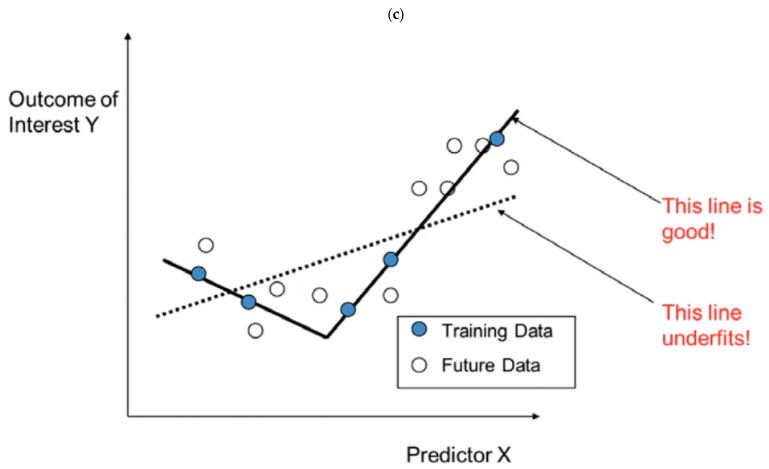
Over- and underfitting in machine learning: (**a**) change in model error during training in under- and overfitting regimes, (**b**) overfitting, (**c**) underfitting. Reproduced under Creative Commons license from ref. [175] with no changes.

The machine learning-based models may use various architectures like convolutional neural networks, recurrent neural networks, multilayer perceptron, graph neural networks, and more. The black-box philosophy of these models makes their usage independent from the knowledge about underlying mechanisms. They scale better with the model size than the ODE-based models; however, on the other hand, they require much more high-quality data to be trained. Another problem is that ML-based models may be easily over- or underfitted to the data (see Figure 29). 

The overfitting is especially likely in cases of multidimensional data with relatively small amount of samples, which is often the case in bioinformatics [175]. In such a case the model does not generalize—it will show much worse performance on a new dataset despite showing great accuracy on training data [175]. We say that the model memorizes the training data, but does not learn the underlying pattern. In the case of underfitting, on the other hand, the model generalizes, but exhibits low performance on both training and testing data [175]. To avoid overfitting, one may use techniques such as cross-validation and regularization. The general idea behind the first strategy is to divide the initial, relatively small training set into k partitions and then train the algorithm k times each time using k − 1 partitions of the initial set as the training data and, at the same time, using the remaining partition as a validation set. Among regularization techniques one can mention the early stopping which relies on breaking the training procedure after detection (using validation data) of early overfitting. Thanks to that the model is not able to memorize the training data. Other regularization techniques involve adding penalty terms to the cost function, as demonstrated in Section 3.1.9 dealing with physics-informed neural networks.

## 4. Summary, Challenges and Future Perspectives

**Figure 30:**
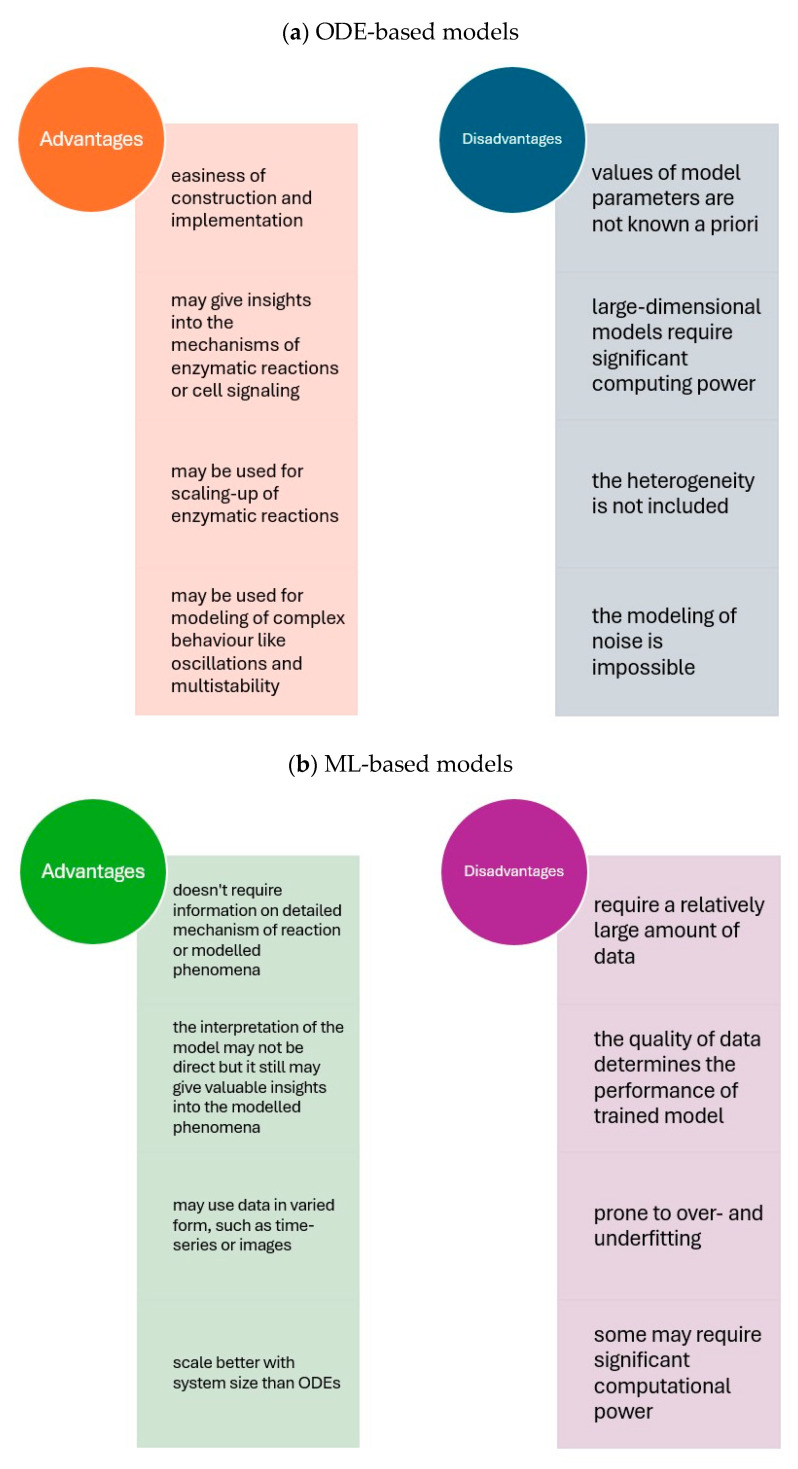
Summary of main advantages and disadvantages of: (**a**) ODE- and (**b**) ML-based models of biochemical reactions and cell signaling.

The summary of main advantages and disadvantages of models based on ordinary differential equations and machine learning is presented graphically as Figure 30.

The future perspectives and challenges for modeling of cell signaling and biochemical reactions may be divided into three main categories:

A. Personalized and precision medicine:
  Using patient-specific data (genetic variants, proteomic profiles) and computational models the prediction of individual responses to various therapies and drugs will likely be available in the future. Such an approach will be especially valuable in the treatment of the autoimmune and cancer diseases.
B. Advancing simulation methods:
  Further improvements in computational methods may be achieved by combining various modeling approaches (so-called hybrid modeling) such as stochastic models, deterministic models, spatial models, machine learning methods and others. Moreover, the utilization of quantum computing may provide exceptional computational sources in terms of the speed of computation.
C. Data integrity and ethical considerations:
  The constantly developing computational models must not violate data protection regulations and privacy. Furthermore, the data utilized in modeling must be standardized to assure their quality.

## 5. Conclusions

Computer-aided modeling enabled simulations and predictions of complex biological phenomena such as cell signaling networks. The role of cell signaling pathways in various diseases may be explained by modeling of their dynamics via different approaches: deterministic (e.g., ODE-based models), stochastic, and machine learning. Further development of computational methods in biology may bring a revolution in fields like drug discovery, personalized medicine, and synthetic biology. Nevertheless, incorporation of such computational methods in real clinical practice demands problem-solving with data standardization, integrity and privacy.

## Data Availability

No new data were created or analyzed in this study. Data sharing is not applicable to this article.
